# A systematic review and meta synthesis of barriers and facilitators to Black women’s participation in healthcare research

**DOI:** 10.1186/s12874-026-02953-7

**Published:** 2026-07-21

**Authors:** S. S. Hall, G. Clancy, S. Patel, N. Thorpe, B. Majmudar, C. Merriman, S. Agyapong, J. Usman, C. Henshall

**Affiliations:** 1 Nottingham Clinical Trials Unit, School of Medicine, University of Nottingham, Nottingham, NG7 2RD UK; 2 University of Nottingham, Jubilee Campus, Wollaton Road, Nottingham, NG8 1BB UK; 3 Library and Knowledge Services, Nottinghamshire Healthcare NHS Foundation Trust, Duncan Macmillan House, Mapperley, Nottinghamshire NG3 6AA UK; 4 Egality Health Ltd., London, SW16 2LP UK; 5 Oxford University Hospitals NHS Foundation Trust, Oxford, England UK; 6 Oxford Health Clinical Research Facility, Warneford Hospital, Warneford Lane, Oxford, OX3 7JX UK; 7 School of Health and Society, University of Salford, Manchester, M6 6PU UK; 8 Oxford Brookes University, Oxford School of Nursing and Midwifery, Jack Straw’s Lane, Oxford, OX3 0BP UK

**Keywords:** Black women, Healthcare research, Clinical trials, Participation barriers, Health equity, Systematic review, Recruitment strategies, Cultural competence

## Abstract

**Background:**

Black women are under-represented in clinical research, contributing to persistent health inequities and undermining the validity and generalisability of research findings. Understanding facilitators and barriers to their research engagement is essential to addressing these challenges. This study aimed to identify and synthesise evidence on barriers and facilitators which influence Black women’s participation in healthcare research, to inform inclusive recruitment strategies and equitable research designs.

**Methods:**

This systematic review was conducted in accordance with PRISMA guidelines and registered with PROSPERO (CRD42024587308). Findings were narratively synthesised using thematic analysis and discussed iteratively through Patient and Public Involvement. Five databases were searched (CINAHL, PsycInfo, Embase, MIDIRS, MEDLINE) for studies published since 2010. Papers in any language were screened for inclusion. Patient and Public Involvement members informed review objectives and data interpretation. Primary research studies reporting on Black women’s experiences, attitudes, and facilitators/barriers to participating in any type of healthcare research. Studies were included if ≥ 50% (to ensure primary representation) of the sample recruited were Black women.

**Results:**

Seventy-two studies were included. Most were from America and spanned research areas relating to oncology, Human Immunodeficiency Virus (HIV), dementia, maternity, and general health. Five overarching themes identified how Black women’s participation in healthcare research is shaped by a complexity of factors. These were *mistrust*, *interpersonal experiences, altruism, self-interest, and low health/research literacy and awareness*. Patient and Public Involvement consultations concurred that the findings aligned with a “bridge and barriers” analogy, illustrating how various factors can facilitate or prevent Black women from engaging with research opportunities. This provided a clear framework to guide culturally sensitive implementation for researchers, healthcare professionals, and policymakers.

**Conclusions:**

Inclusive strategies for recruiting Black women into research must focus on co-constructing a bridge between communities and research systems. This requires structural and systemic change in research delivery environments and ongoing collaborations with all stakeholders including Black women, healthcare system providers, and research teams.

**Trial registration:**

PROSPERO (CRD42024587308).

**Supplementary Information:**

The online version contains supplementary material available at https://doi.org/10.1186/s12874-026-02953-7.

## Background

Health inequities affecting Black women highlight the importance of ensuring their needs are understood within healthcare research distinct from other minoritised ethnic groups. inequities faced by Black women are evident across a range of health outcomes. For example, Black women are three to four times more likely to die from pregnancy-related causes than White women [1–3]. They are also at a higher risk of giving birth prematurely; this increases risks relating to infant mortality and development [1–3]. Black women have higher incidences of multiple chronic conditions compared to White women, including hypertension, osteoarthritis, diabetes, obesity and cancer [4–7]. Despite having a lower breast cancer incidence than White women, Black women are more likely to die from breast cancer, and have higher rates of cervical cancer [8, 9]. These wide-ranging inequities are driven by factors including systemic racial bias across healthcare services, limited access to these services, pre-existing health conditions [6], delayed diagnosis and inadequate preventative care. Additionally, cultural factors, such as reliance on intergenerational knowledge, may reduce engagement with healthcare services [10–12]. Discrimination is also a factor affecting health outcomes, for example 65% of Black people in the United Kingdom (UK) reporting feeling discriminated against by health care professionals responsible for their care on the grounds of ethnicity [13].

Addressing the above inequities requires greater inclusion of diverse ethnic groups in research to ensure that healthcare treatments and their delivery are effective and applicable to all populations [14]. Genetic, environmental, and cultural differences can affect how people respond to pharmacological and non-pharmacological treatments, meaning that research based solely on homogenous groups may lead to biased results and unequal outcomes [15, 16]. However, Black women are frequently underrepresented in clinical research [17], with rates of participation in neuroscience (1.3%), cancer (7.3%) and COVID-19 prevention trials (7.2%) disproportionally low compared to national population statistics [18–21]. Internationally, mistrust in healthcare research among Black communities, and particularly Black women, is rooted in a history of systemic racism, exploitation, and marginalisation. Examples such as the Tuskegee Syphilis Study, Henrietta Lacks and the legacy of forced sterilisations and reproductive control policies targeting Black women [22–24] exemplify this.

This long-established mistrust extends to contemporary healthcare research, with Black women commonly expressing concerns during the research process about unequal treatment, a lack of culturally sensitive communication, and poor relationships with healthcare professionals and clinical researchers [25–27]. Clinical research teams, in particular healthcare professionals in research delivery roles, are often responsible for recruiting to healthcare research and are well placed to promote participation to Black women. However, these staff report needing greater cultural competence, including tailored training and resources to support effective and sensitive discussions about research participation with Black women [28, 29]. Evidence suggests that African American patients receive less time being provided with information about clinical research than other population groups [30]. This reflects not only systemic inequities in healthcare research, but also a lack of confidence among some research teams about how to communicate inclusively and respectfully with diverse patient groups. Existing reviews have examined barriers and facilitators to research participation within ethnic minority communities more broadly; however, few have focused specifically on Black women [26, 31]. Reported barriers include poor relationships with medical and research staff, the financial burden of participation, and personal circumstances [26, 31], but there is a need to identify and better define these challenges, specific to Black women [31]. The fragmentation of our understanding across ethnic groups and methodological approaches makes it difficult to draw coherent conclusions or translate insights into practice. A clearer, more coherent evidence base is crucial at a time when the NHS 10 Year Health Plan explicitly prioritises efforts to reduce longstanding health inequities [32].

A systematic synthesis provides a rigorous means of consolidating existing evidence, identifying consistent themes, and highlighting remaining uncertainties [33]. Such clarity is essential for informing targeted recruitment strategies, guiding policy development, and supporting the design of equitable and inclusive research. Therefore, the present systematic review aimed to identify and analyse barriers and facilitators to Black women’s participation in healthcare research. Secondary aims were to determine factors influencing research retention, assess how different healthcare settings shape participation, and examine the role of wider societal influences on Black women’s decision-making around research involvement.

## Methods

The systematic review was undertaken in accordance with the Preferred Reporting Items for Systematic Reviews and Meta-analyses (PRISMA) guidelines [34]. It was registered on the International Prospective Register of Systematic Reviews (PROSPERO) protocol [CRD42024587308]. The paper has been written in accordance with the PRISMA-Equity Checklist (Additional file 1).

### Search strategy

The search strategy was developed by the study team, with support and guidance from their organisation’s library services (Additional file 2). EBSCOhost CINAHL Plus with Full Text, Ovid PsycInfo, Ovid Embase, MIDIRS (Maternity & Infant Care Database) and Ovid MEDLINE ALL databases were searched in September 2024 and updated in December 2025. Studies published since 2010 were included, aligning with the implementation of the UK Equality Act 2010, which marked an important policy shift in relation to equity and non-discrimination in research and healthcare. While this review includes studies from multiple international contexts, the Equality Act was used as a pragmatic temporal anchor, reflecting the study’s UK-based perspective rather than implying global policy alignment.

Papers published in all languages were eligible for inclusion. The Rayaan platform [35] was used to manage data from the systematic review. The review team (SSH, CH, SP, GC, SA, CM, JU) double screened titles and abstracts and a third reviewer from the team was consulted regarding any selection discrepancies. Following this, all full texts remaining were double screened for eligibility.

### Inclusion criteria

Studies were eligible for inclusion if they met the following criteria:

- Population: Reported on Black women (of any age) considering or engaging in participation in healthcare research.
- Phenomenon of interest: Examined attitudes, willingness, barriers, facilitators, or decision-making related to participation, recruitment, or retention in healthcare research.
- Study characteristics: Empirical primary research using any study design (qualitative, quantitative, or mixed-methods).
- Context: Any healthcare or research setting, regardless of the detail provided on this.
- Geographical scope: No restrictions.

### Exclusion criteria

- Publication type: Literature reviews, systematic reviews, conference abstracts, study protocols, or unpublished website material without identifiable authorship or formal output.
- Population clarity: Did not clearly distinguish data relating to Black ethnic groups, or included samples in which fewer than 50% of participants were Black women (to ensure primary representation).
- Date of publication: Published before 2010.
- Availability: Full text not accessible after contacting the author and librarian support.

### Data extraction

Using a pre-defined data extraction tool (Additional file 3), study characteristics and key findings data were extracted from all included papers by one author (GC). In addition, 20% of included papers were verified for accuracy of data extraction by another team member (SA, CM, JU), no substantive discrepancies were identified. Information extracted included paper authors and year of publication, country study conducted in, healthcare topic, participant recruitment information, study aim, study focus (recruitment and/or retention), and study design.

### Outcomes of interest

- Primary outcome: Factors influencing the recruitment of Black women into healthcare research, to identify important factors to inform more equitable recruitment processes.
- Secondary outcome: Factors influencing the retention of Black women in healthcare research, to support more equitable participation and better understanding of Black women’s experiences. Factors of interest included cultural, environmental (e.g., healthcare setting), and social influences (e.g., roles of family members, community networks, and healthcare professionals).

### Synthesis of results

The synthesis approach used to analyse and interpret the data was informed by Noblit and Hare’s work on meta-ethnography and developed into thematic-synthesis by Thomas and Harden [30, 36]. The synthesis was led by GC (expertise in qualitative maternal health research) with supervision and support from SSH, CH and SP (expertise in systematic reviews and conducting healthcare research). The process consisted of three iterative stages: (1) line-by-line coding of text (first-order constructs); (2) developing descriptive themes while remaining close to the primary text (second-order constructs); and (3) generating analytical themes by adding new interpretative constructs and hypotheses in relation to the original research question (third-order constructs) [36]. A thematic synthesis approach was selected because it enabled the integration of evidence from qualitative, quantitative and mixed-methods studies, whilst retaining participants’ perspectives and supporting the development of higher-order analytical interpretations across diverse healthcare contexts.

Papers were initially read to support familiarisation with the data and subsequently revisited during coding to extract first-order constructs using NVivo 12, identifying similarities and differences between codes to develop second-order descriptive themes. These initial groupings were discussed with SSH prior to the compilation of third-order constructs. Codes were generated inductively from study findings and compared across studies to identify recurring patterns, similarities, and differences. Descriptive themes were developed through constant comparison and subsequently refined into higher-order analytical themes that explained relationships between barriers and facilitators influencing research participation. This process, and the emerging findings, were iteratively discussed with the team to support the reflexive practice of mutual collaboration, where cycles of dialogue help move understandings beyond initial assumptions, understandings and biases [37]. Key findings were reviewed and discussed with Patient and Public Involvement (PPI) representatives and a community engagement organisation to ensure the findings resonated with those with lived experience, and to support wider understanding and implementation amongst a range of stakeholders.

### Patient and Public Involvement (PPI)

Eleven mothers who identified as women of Black ethnicity formed the study PPI group, along with a representative from Egality Health (author BM), a leading community engagement agency. All PPI meetings were conducted online at times to suit the members of the group. A trauma-informed facilitation approach was used to ensure sensitivity to the inequities experienced by Black women. The PPI group contributed to shaping the review objectives, ensuring alignment with priorities relevant to Black women’s experiences of healthcare and research. They were also actively involved in the analysis of findings, including reviewing data codes derived from the thematic synthesis during a PPI consultation meeting. Key review findings were translated into a series of scenarios to promote comprehension, and PPI members were asked to explore their likely responses to participation in each, alongside suggesting ways to improve these experiences. This input supported the ongoing refinement and interpretation of themes. We also examined the extent to which emerging findings aligned with PPI perspectives, providing an additional layer of triangulation. PPI discussions were instrumental in shaping the conceptual framing of the findings as a ‘bridges and barriers’ model, with participants reflecting on how different factors either enabled or hindered movement between communities and research systems. Finally, PPI representatives contributed to discussions around the implications and recommendations, directly shaping the development of the “bridges and barriers” conceptual framework and supporting its relevance and applicability to real-world contexts. A further paper is being prepared which details the contributions and lessons learned from working closely with PPI members throughout this review and the wider study associated with it.

### Quality assessment

A quality review of the research was conducted by one author (GC), with 20% of papers independently checked by a second author (SSH) using the Critical Appraisal Schemes Programme (CASP) tools and/or the Mixed Methods Appraisal Tool (MMAT). Where there was disagreement, consensus was reached through discussion between the two authors, without the need for a third reviewer. Studies were not excluded on the basis of quality because the review aimed to capture the breadth of evidence relating to Black women's participation in healthcare research. Instead, appraisal findings were used to inform interpretation of the evidence and consideration of the relative contribution of studies during synthesis [38].

## Results

### Description of studies

**Fig. 1:**
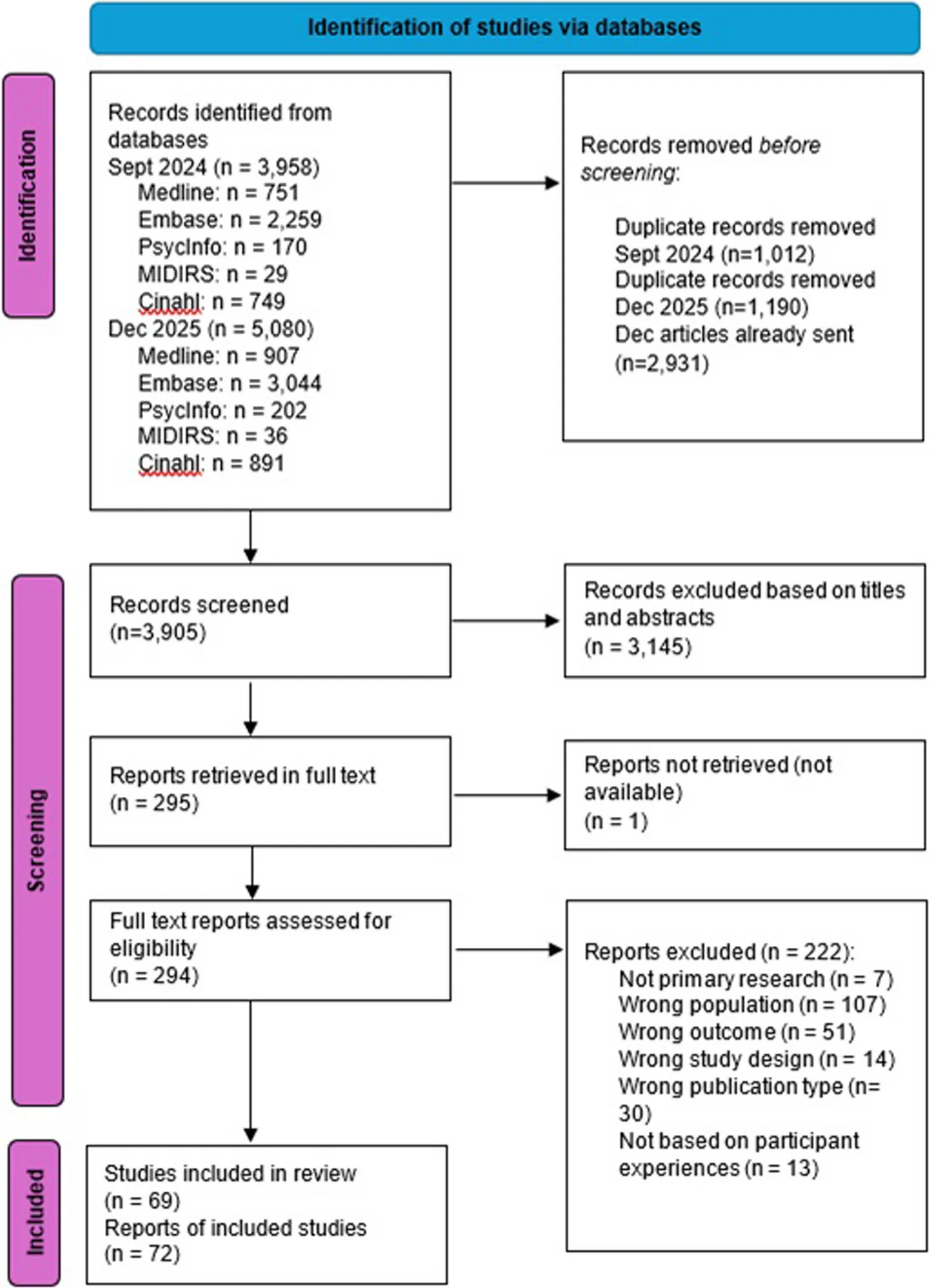
PRISMA

The original database search in September 2024 yielded a total of 3,958 records. After the removal of duplicates and screening of titles, abstracts, and full-text papers, 60 papers were included in the review (Fig. 1). The updated database search in December 2025 yielded 5,080 records and after the removal of duplicates, papers previously screened, and the screening of new papers, 12 papers were added to the review. Summary characteristics of the included papers are shown in additional file 4.

The 72 empirical papers included mixed-methods (*n* = 16) [25, 39–53], quantitative (*n* = 18) [54–71] and qualitative (*n* = 38) studies [72–109]. Most studies were undertaken in the USA (*n* = 64), with some from Canada (*n* = 4) [40, 71, 77, 83], Nigeria (*n* = 2) [53, 91], South Africa (*n* = 1) [59] and the UK (*n* = 1) [104]. The majority focused on the experiences of African Americans, however nine studies specifically focused on experiences of immigrant Black women [40, 42, 50, 63, 66, 77, 83, 85, 86]. Terminology used to describe participants varied across studies and reflected local sociocultural contexts. Most studies conducted in the United States used the term "African American", while studies from Canada, the UK, and African settings more commonly referred to “Black", "African”, “Caribbean" and "Black/African immigrant". For consistency, this review uses the term "Black women" as an umbrella descriptor while recognising the heterogeneity of identities, migration histories, cultural backgrounds, and experiences represented across the included studies.

Nineteen studies recruited only Black women [44, 47, 51, 59, 60, 62, 65, 67, 74, 77, 80, 82, 83, 87, 93, 98, 102, 107, 108], and one recruited only Black transgender women [90]. All studies focussed on issues relating to the recruitment of Black women to research, but only nine focussed on retention issues [47, 53, 54, 58, 60, 75, 76, 78, 109].

Research in oncology was the most common healthcare specialty (*n* = 21) [25, 41, 42, 44, 47, 53, 54, 57, 60, 63, 67, 69, 76, 79, 81, 87, 93, 94, 97, 107, 108], followed by Human Immunodeficiency Virus (HIV) (*n* = 10), [51, 56, 59, 61, 65, 71, 75, 83, 90, 98] general research participation (*n* = 13) [48, 50, 52, 64, 68, 73, 85, 86, 91, 102, 103, 105, 109], and Alzheimer’s/dementia (*n* = 6) [43, 72, 78, 84, 92, 99]. One paper focussed on maternity care [45]. The healthcare setting in which the studies took place was poorly reported (for example, whether they were conducted in community, primary, or secondary healthcare environments). However, a small number of studies were identified as seemingly taking place in community settings (*n* = 6) [42, 52, 57, 86, 101, 106], secondary care (*n* = 3) [53, 107, 108] primary care (*n* = 1) [45], or primary and secondary care (*n* = 1) [66].

### Quality assessment

All included studies were assessed as being of adequate methodological quality and no concerns were raised in relation to bias.

56 studies were assessed using CASP tools. Studies were generally of good methodological quality, with most demonstrating clear aims, appropriate study designs, robust data collection methods, and transparent reporting of findings. Methodological strengths and limitations varied across study designs. However, qualitative studies generally provided greater conceptual and experiential detail, which enabled a richer contribution to theme development. These observations reflect differences in data richness rather than direct comparisons of methodological quality across study designs. The most common limitation across quantitative studies was a lack of information regarding sample size adequacy, transferability, and explanatory depth, and for qualitative studies it was limited consideration of researcher reflexivity and positionality on the research process.

16 studies were assessed using MMAT. Overall, these studies demonstrated clear research questions, appropriate study designs, and data collection methods aligned with their stated aims. Common methodological limitations included uncertainty regarding sample representativeness and the potential for non-response bias in quantitative descriptive studies, alongside variable integration and interpretation of findings within mixed-methods research. A small number of studies provided insufficient methodological detail for some appraisal criteria to be confidently assessed, although no study was judged to be of sufficiently poor quality to warrant exclusion.

The analytic utility of the evidence was primarily determined by the conceptual and experiential richness of the data – that is, the extent to which studies provided detailed, nuanced accounts of Black women’s experiences [110]. As such, qualitative studies providing detailed accounts of Black women's experiences contributed the most to the development of themes. Quantitative and mixed-methods studies generally offered more limited depth, constraining their capacity to elucidate the underlying mechanisms and contextual factors shaping Black women’s participation, or non-participation, in healthcare research. No studies were excluded for being of poor methodological quality.

### Key themes

**Fig. 2:**
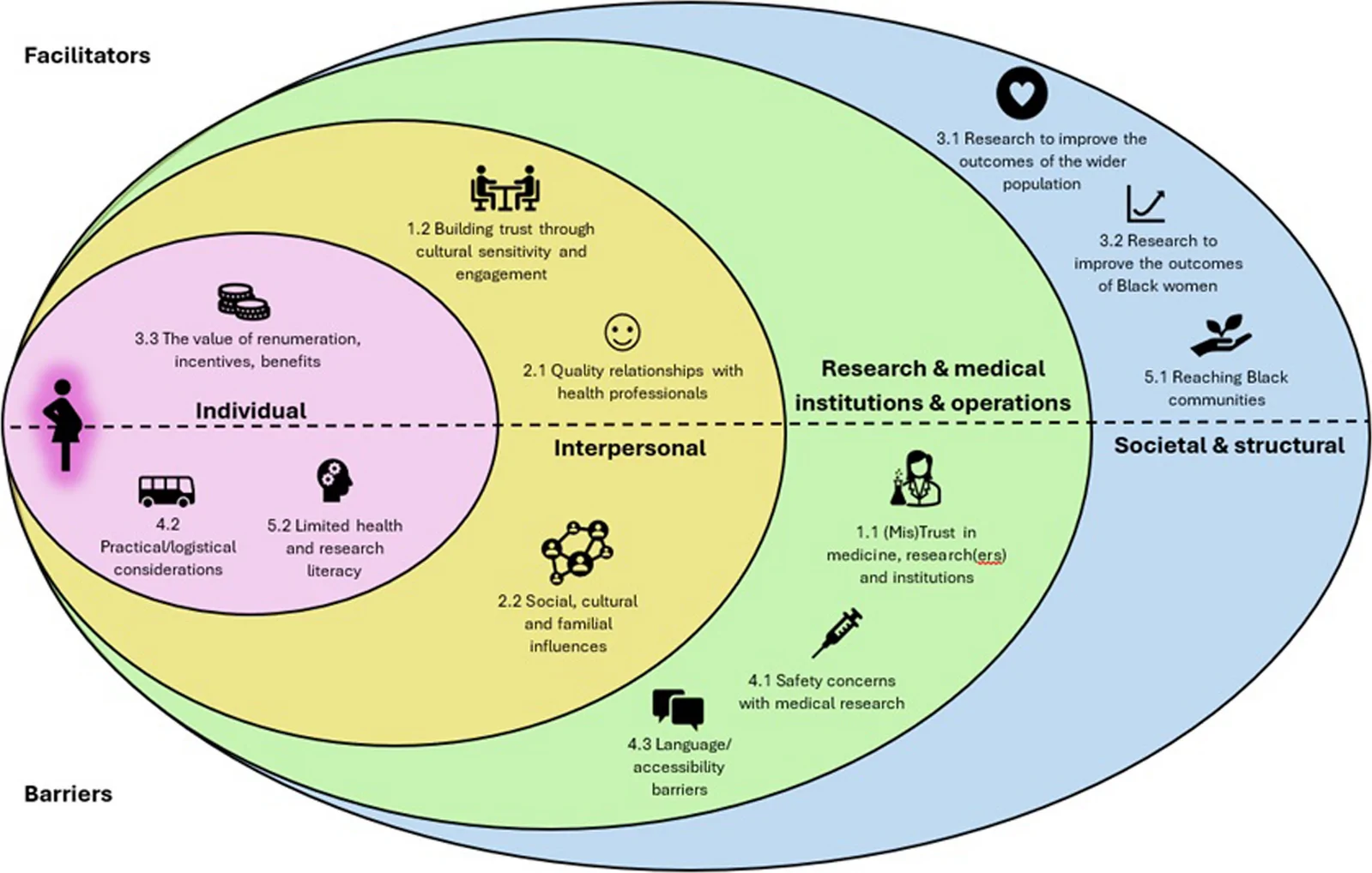
Contextual overview of findings

Five themes were generated; these were: (1) Historical and Contemporary Drivers of Mistrust in Research Participation; (2) Social and Relational Determinants of Engagement; (3) Altruism and Personal Interest as Drivers of Research Participation; (4) Weighing the Burdens and Risks of Research Participation; and (5) Structural and Informational Foundations of Research Participation (Table 1, Fig. 2). Each theme contained both barriers and facilitators to participation, reflecting the dynamic ways in which similar domains (for example trust, relationships, or information) could either hinder or enable engagement depending on how they were experienced. More detail on each theme and their relevant subthemes is provided below and additional file 5 provides detail of the evidence underpinning each theme/sub-theme with supporting quotes.

**Table 1:** Main themes and sub-themes

Main themes/Domains	Sub-themes
1. Historical and Contemporary Drivers of Mistrust in Research Participation	1.1 (Mis)Trust in medicine, research(ers) and institutions Building trust through cultural sensitivity and engagement
2. Social and Relational Determinants of Engagement	2.1 Quality relationships with health professionals 2.2 Social, cultural and familial influences
3. Altruism and Personal Interest as Drivers of Research Participation	3.1 Research to improve the outcomes of the wider population 3.2 Research to improve the outcomes of Black women 3.3 The value of remuneration, incentives, benefits
4. Weighing the Burdens and Risks of Research Participation	4.1 Safety concerns with medical research 4.2 Practical/logistical considerations 4.3 Languages/accessibility barriers
5. Structural and Informational Foundations of Research Participation	5.1 Reaching Black communities 5.2 Limited health and research literacy

## Theme 1. historical and contemporary drivers of mistrust in research participation

This theme highlighted the multifaceted aspects of (mis)trust that Black women often hold towards research participation. However, the data also identified opportunities to build trust through meaningful engagement.

### (Mis)Trust in medicine, research(ers) and institutions

Forty-five studies were classified within this theme. Many studies reported deep-rooted mistrust of healthcare systems, professionals and research among Black communities, shaped by both personal experiences and collective historical trauma—including well-known cases like the Tuskegee experiments and Henrietta Lacks [48, 56, 63, 71, 73, 89, 94, 99, 101, 102]. This mistrust extended beyond healthcare to include government and political institutions that fund and regulate medical interventions [39, 72, 94], and was sometimes rooted in broader scepticism toward science, favouring spiritual beliefs (e.g. ‘God’s will’) [25, 47, 50, 66, 97] or natural remedies [39, 79]. These alternative understandings and approaches appeared to function, at least in part, as responses to concerns about the trustworthiness, motives, or safety of medical research, rather than purely social or cultural influences. Studies described participant fears of being treated as “guinea pigs,” suspicion that treatments might be designed to harm Black people, and racially biased care [39, 40, 44, 46–48, 50, 52, 72, 74, 78–81, 84, 89, 93, 94, 96–99, 104, 105]. These concerns, grounded in both generational knowledge and lived experience, significantly shaped Black women’s willingness to engage in health research, despite some researchers’ efforts to counteract these narratives [85].

Mistrust was also directed towards research processes and protections, particularly around researcher competency and ethical conduct [43, 90]. Participants expressed concerns about impersonal recruitment methods and feared that studies might prioritise research outcomes over participant safety and care [40, 44, 48, 59, 79, 93, 94, 98, 100]. Scepticism about researchers’ motivations, such as financial gain or fulfilling policy requirements, undermined trust in their genuine interest in the health and wellbeing of Black communities [47, 74, 81, 85, 89, 93]. Additionally, fears around data privacy and misuse, such as personal information being leaked or used beyond the scope of the study, were frequently cited [25, 40, 43, 62, 77, 78, 82, 83, 94, 99, 100, 102, 104, 105].

### Building trust through cultural sensitivity and engagement

Thirty-six studies highlighted key factors identified for building trust through cultural sensitivity and engagement. These included the provision of professional, accessible, transparent and detailed information about the research (including ethics/participant protections) and researchers [44, 47, 49, 53, 71–74, 78, 79, 83, 85, 86, 103, 109]. Cultural competency, awareness of health inequities and assurances about confidentiality were reported to help put participants at ease, making them feel safe, valued and respected [42, 46, 65, 72, 77, 80, 82, 85, 86, 99].

Most studies reported that Black women valued continuous and meaningful community engagement when considering research participation (including dissemination of results) [44, 50, 52, 57, 58, 63, 72–74, 78, 87, 92, 96, 99, 102]. They were also more likely to consider taking part if the study had been endorsed or recommended to them by a trusted source (e.g. a known doctor or community/religious organisation/leader) [47, 50, 53, 56, 73, 80].

## Theme 2. social and relational determinants of engagement

The quality of interactions with research delivery teams and healthcare professionals more broadly were found to be key to influencing the recruitment and retention of research participants. Friends, family members and communities could also influence a potential participant's awareness of research and decisions to take part.

### Quality of relationships with health professionals

Thirty-seven studies discussed the importance of relationships with healthcare professionals. Positive interactions with research delivery teams, including administrative staff, were reported to have had an important influence on participants engagement and experiences. In particular the importance of developing trusting relationships with friendly, enthusiastic, knowledgeable and professional members of the research team was recognised [40, 53, 54, 72, 73, 75, 78, 85, 86, 88, 89, 93–96, 99, 102, 108, 109]. Conversely, negative interactions with study teams where individuals felt rushed or ‘pushed’ into taking part were often perceived as impersonal, disingenuous or disrespectful [75, 86, 99].

Authentic, rather than tokenistic, diversity in research teams could also help to build research trust amongst Black communities. Factors associated with authentic diversity included Black researchers in prominent positions and the gender of the researcher [25, 42, 44, 62, 72–74, 77, 78, 80, 81, 83–85], as well as sharing a language [85] or having personal experience of the study topic area [95]. Studies showed that diversity in the research team could be particularly important for research on sensitive or stigmatised health topics (e.g. domestic violence, reproductive/sexual health) or where participants feared that they or their communities might be judged or stereotyped as a result of the research [80, 95].

Some participants felt reassured when researchers or healthcare professionals demonstrated cultural competence [25, 96]. However, concordance between researcher and participant such as shared ethnicity was not universally seen as necessary or sufficient for building trust. In fact, one study highlighted that Black researchers had historically been complicit in unethical practices, such as the Tuskegee experiments, which undermined assumptions about automatic trust based on shared identity [79]. Another study highlighted concerns that researchers from their own communities might compromise anonymity or confidentiality [83]. Instead, the researchers’ integrity, transparency, and respectful qualities that could transcend ethnic or cultural differences and foster trust were found to be important [53, 85, 102]. Positive research experiences were also shown to improve trust in medicine and encourage wider healthcare-related engagement [68, 86].

### Social, cultural and familial influences

Social factors were mentioned in 40 studies. Hearing about research from a trusted source, whether a known healthcare professional, community organisation (especially churches) or prominent community figure was reported to influence research participation (both recruitment and retention) in both directions, depending on the viewpoint of that figure [40–42, 44, 47, 52, 54, 56, 61, 63, 71–73, 75, 80, 81, 83, 87, 88, 99, 103, 105].

Discussing study participation with family members was identified as a key factor in determining participation, even if the decision ultimately still lay with the individual [25, 41, 44, 49, 62, 81, 93, 96, 98, 101, 105]. Two studies conducted in Nigeria highlighted that culturally, women had limited autonomy and would need to obtain permission from their husband/family head’s to take part in research studies [53, 91].

Cultural norms emerged as barriers to participation in some studies, where, in Black communities discussing personal matters was seen as unconventional and discouraged by a prevailing ‘code of silence’ [50, 74, 77]. Concerns about stigma were also prominent especially when research involved sensitive or potentially controversial health topics [59, 65, 89, 95]. In some instances, cultural beliefs further complicated engagement, with some individuals expressing fear that providing biological samples for research could expose them to spiritual harm or bewitchment [83].

## Theme 3. altruism and personal interest as drivers of research participation

Studies showed that participants were often motivated to take part in research because they viewed it as beneficial to wider society, whilst also improving the lives of Black women specifically, and offering personal benefits including financial and non-financial gains.

### Research to improve the outcomes of the wider population

Thirty-six studies reported the importance of contributing to the wider population, and that altruism was the key motivator for taking part in research [25, 39, 43–45, 48, 50–52, 62, 66, 67, 69, 71–74, 82, 83, 90, 93, 95, 96, 99, 103–105, 107]. This act of service could be further enhanced if individuals had a personal connection to the condition under study [25, 48, 61, 62, 64, 67, 78, 89, 98]. However, participants’ altruistic approach was generally balanced against the personal risk involved with taking part [41]. The potential to contribute to science and society through research was highlighted as an important benefit that needed to be more clearly communicated to potential participants during recruitment [73, 87].

### Research to improve the outcomes of Black women (*n* = 23)

Twenty-three studies commented specifically about research benefits for Black women. There was a strong desire to increase the representation of Black women in research to ensure their voices were heard and that treatments were developed that were inclusive of their specific needs and experiences [67, 71–73, 77, 78, 80, 97, 102, 107]. Several studies stated that Black women wanted to know that the research they were contributing to would benefit; other Black women, Black communities in general, women of other ethnicities, and those with the same health condition [45, 46, 51, 57, 62, 74, 76–78, 80, 94–97, 99, 106]. Understanding who stood to benefit from the research was identified as crucial for Black women to feel that their participation was meaningful [87, 94].

### The value of remuneration, incentives, benefits

Thirty-four studies found that participants were also motivated to take part in research studies due to the perceived benefits of doing so. For some these benefits were financial, such as monetary incentives, but also included reimbursement for the costs of participation (e.g. travel, childcare) [39, 42, 43, 48, 52, 53, 62–64, 71, 74, 75, 87, 89, 92]. However, perceived benefits also included early access to treatments [52, 59, 62, 93], gaining knowledge or skills to improve health [44, 45, 48, 60, 62, 71, 74–76, 78, 90, 95, 98, 100, 101, 104–106, 109], engaging and socialising with others [95, 98, 109] and therapeutic experiences related to participation [51, 75, 82, 95, 98].

## Theme 4. weighing the burdens and risks of research participation

Those seeking to take part in medical research were often looking to minimise the burden of participation. The burdens associated with participation were varied and include practical, physical and psychological/emotional considerations.

### Safety concerns with medical research

The primary burden of participation—highlighted by 32 studies—related to safety concerns, including potential side effects or unexpected consequences of taking part. For some, the potential side effects of testing new healthcare treatments seemed more prominent than the potential benefits. Participants expressed concerns due to fear of the unknown, concerns about being given a placebo, and how new treatments might interact with their current medications [25, 39, 41, 48, 52, 53, 56, 59, 71, 72, 79, 81, 89, 90, 92, 93, 96, 98, 99, 101].

Several studies stated that participants perceived that a potential concern about taking part in healthcare research could be discovering ill-health or a predisposition to a condition that they were previously unaware of, and the psychological burden this would bring [25, 41, 72, 78, 83, 93, 95, 98]. Some studies reported that Black women felt more comfortable taking part in research that was non-invasive or observational/behavioural in nature (perceived as low risk) compared to clinical/medical research involving treatments or procedures (perceived as high risk), especially for pregnant women and their unborn children [44, 49, 70, 92, 98, 99, 102, 104–107].

### Practical/logistical considerations

Another key burden of participation—reported by 29 studies—related to the practicalities of taking part. These included: the time commitment involved, particularly for longitudinal studies [25, 40, 42, 50–52, 54, 62–64, 71, 75, 87, 92, 95, 99, 109], as well as the actual time of participation with out-of-hours options deemed beneficial location and travel time involved with home visits or mutually agreed community-based locations often considered helpful [25, 40, 44, 46, 50, 57, 59, 70, 81, 89, 90, 92, 95, 98, 99, 103, 104, 109], lifestyle disruptions, including interrupting care responsibilities or needing to take time off work [25, 89, 98], and a lack of digital resources for online/remote studies [46, 62].

### Languages/accessibility barriers

Language and accessibility were reported in ten papers as barriers to research participation. A lack of high-quality interpretation and clear, accessible materials in different languages and formats (e.g. physical copies, online resources, videos) were reported as key barriers to attracting a diverse sample [44, 50, 63, 87, 94, 95, 99, 102, 103]. Some studies also reported the benefits of giving women sufficient time to read, reflect and ask questions about their participation as part of the informed consent process, checking they understood *what* the study was about and *why* they were being invited to take part [87, 96].

## Theme 5. structural and informational foundations of research participation

Low levels of health literacy as well as low awareness of (medical) research could inhibit potential participant's understanding of why and how they should or could take part in studies. Having improved levels of knowledge enabled individuals’ sense of candidacy for both research and healthcare more broadly.

### Reaching Black communities

Eleven studies noted a widespread lack of awareness about research opportunities and how individuals could get involved [39, 43, 46, 50, 57, 64, 73, 84, 89, 93, 96]. The reviewed evidence revealed a wide range of recruitment strategies, with several identified as particularly effective in engaging Black women participants in particular, including: leaflets/flyers, in-person recruitment at medical settings, in-person recruitment at health fairs, word-of-mouth, community and religious organisations/networks, mass-mailing, social media and e-recruitment (e.g. text messages, emails, websites) [39, 40, 44, 52, 54, 55, 80, 88, 107, 108]. Whilst mass media was felt to be good for awareness raising, it was considered an ineffective tool for recruitment because of its impersonal nature [40, 47].

### Limited health and research literacy

Twenty-seven studies reported that a key barrier to Black women taking part in research was a lack of knowledge about medical research/clinical trials. This included a lack of; understanding of research designs and methodologies [72], research processes and jargon [25, 41, 48, 53, 56, 71, 73, 74, 78, 79, 81, 93, 97, 101, 109], and assurances about the safety of the intervention being tested [25]. It was recognised that efforts were needed to increase health literacy and educate populations on research, health conditions and treatments to increase research accessibility [39, 44, 72, 89, 102] and potential future participation [78, 96].

### Recommendations

**Fig. 3:**
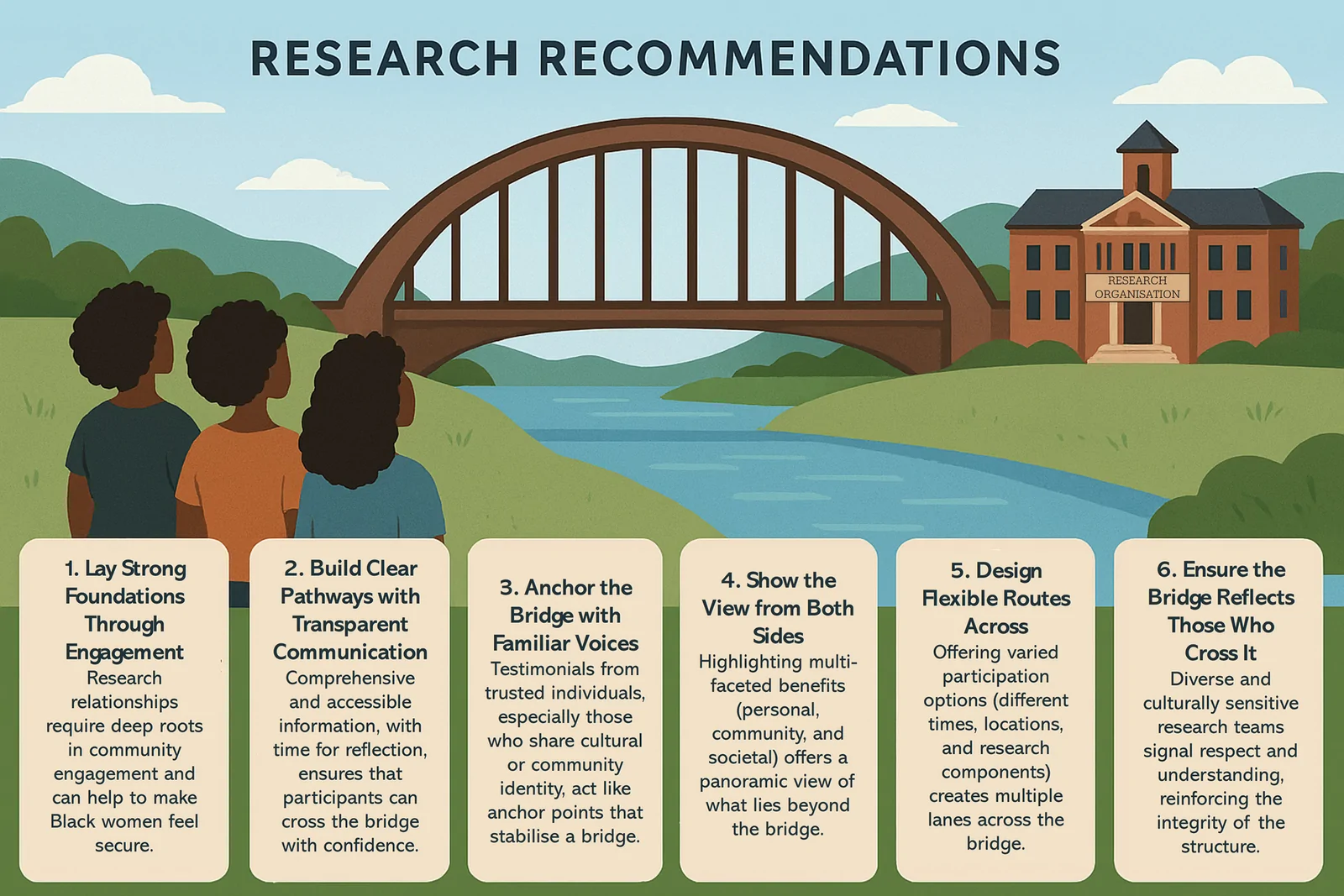
Bridges and barriers infographic

The barriers and facilitators identified in this review highlight that improving Black women’s participation in healthcare research requires more than recruitment strategies; it necessitates systemic change. Drawing on lived experience feedback, from Black women and Research Delivery Teams, we have developed a bridges and barriers analogy to support a conceptual understanding of these findings (Fig. 3). The Bridges and Barriers conceptual framework, emerging from PPI discussions, offers a novel and practical lens for understanding how these diverse influences collectively shape participation. Within this framework, participation is conceptualised as a crossing between Black communities and research systems, the framework foregrounds the structural conditions that either facilitate or obstruct engagement. Each factor identified in the review either reinforces the bridge, making the crossing safer, more accessible and more welcoming, or acts as a barrier by weakening the structure, narrowing the pathway, or obstructing access altogether. Additional file 6 provides examples of 1 st and 2nd order constructs and their corresponding 3rd order constructs.

Table 2 summarises recommendations developed by the study team, including PPI members, based on the review findings in relation to the ‘Bridges and Barriers’ conceptual understanding above. Examples of practical application are provided to support translation into research practice. As summarised in Table 2, strengthening this “bridge” requires foundational investments in trust, transparency, flexibility, and cultural humility, alongside tangible equity-oriented measures such as accessible study designs, fair compensation, and reduced participant burden. Reducing participant burden and improving health literacy is critical for recruitment and retention, particularly in decentralised or remote trials.

**Table 2:** Recommendations for recruiting and retaining black women informed by the literature

Recommendation 1: Lay Strong Foundations Through Engagement [43, 44, 47, 50, 54, 61, 67, 68, 72, 78–81, 83, 86, 92, 95, 96, 99]
Just as a bridge needs solid foundations, research relationships require deep roots in community engagement. Sustained visibility, such as attending community events or hosting educational workshops, reinforces trust and demonstrate the value of research in spaces where Black women already feel secure. This can help participants to feel ownership of the process, strengthening the bridge for both recruitment and retention
Example: Attend church events; host community Q&A sessions; partner with local organisations
Recommendation 2: Build Clear Pathways with Transparent Communication [43–45, 48, 50, 51, 54, 65, 81, 86, 87, 89, 90, 92, 93, 96, 99, 100]
A fragile bridge becomes safer when its structure is clear and predictable. Similarly, providing plain-language, culturally sensitive information about study details, risks, benefits, and processes before consent ensures participants can cross with confidence. Giving time for reflection within their community strengthens trust and reduces uncertainty
Example: Provide plain-English leaflets; explain risks upfront; allow time for questions
Recommendation 3: Anchor the Bridge with Familiar Voices [39, 44, 48, 52, 54, 72, 73, 75, 78, 87, 90, 93, 95, 99]
Testimonials act like anchor points that stabilise a bridge. Hearing from trusted individuals, especially those who share cultural or community identity, can reassure potential participants that the crossing is safe and worthwhile. These voices help transform hesitation into confidence
Example: Use community champions; share participant testimonials; involve trusted healthcare professionals
Example: Use community champions; share participant testimonials; involve trusted healthcare professionals
Highlighting multi-faceted benefits (personal, community, and societal) offers a panoramic view of what lies beyond the bridge. When participants see how their involvement contributes to broader well-being, the crossing feels purposeful and rewarding
Example: Explain personal and community benefits; show how findings will be used
Recommendation 5: Design Flexible Routes Across the Bridge [44, 45, 50, 54, 72, 83, 99, 100]
Not all travellers cross the same way. Offering varied participation options (different times, locations, and research components) creates multiple lanes across the bridge. This flexibility respects individual circumstances and preferences, making participation more accessible and less burdensome
Example: Offer evening appointments; provide childcare support; enable remote participation
Recommendation 6: Ensure the Bridge Reflects Those Who Cross It [44, 72, 80, 81]
A bridge feels safer when it resonates with those who use it. Diverse and culturally sensitive research teams signal respect and understanding, reinforcing the integrity of the structure. While matching identities can help, the deeper strength lies in inclusive practices and strategies that honour participants’ lived experiences
Example: Include diverse research staff; co-design materials; reflect cultural perspectives
Recommendation 7: Strengthen Community and Family Pathways to Participation
Research participation decisions are often made within broader family, social and community contexts. Researchers should recognise the influence of trusted family members, peers, faith leaders and community organisations, and provide opportunities for participants to discuss research with those important to them

## Discussion

### Statement of principal findings

This systematic review synthesised evidence from 72 studies to identify the multifaceted barriers and facilitators influencing Black women’s participation in healthcare research. Participation was primarily considered in the literatures in terms of recruitment, with limited attention paid to retention. Five overarching themes emerged: (1) Historical and Contemporary Drivers of Mistrust in Research Participation, (2) Social and Relational Determinants of Engagement, (3) Altruism and Personal Interest as Drivers of Research Participation, (4) Weighing the Burdens and Risks of Research Participation, and (5) Structural and Informational Foundations of Research Participation. Mistrust, rooted in historical and contemporary experiences of racism and exploitation, was the most frequently cited barrier, while meaningful community engagement and culturally competent communication were key facilitators. The review highlighted that participation was shaped not only by individual-level factors but also by structural and relational dynamics that intersect with race, gender, and socioeconomic status. This includes social influences such as discussing participation with family members or hearing about research through the community, as well as the diversity, cultural competence and integrity of healthcare professionals/research delivery teams. In most of the literature, healthcare setting (e.g., primary, secondary) was unclearly reported and therefore it was not possible determine if it made a difference to participants’ willingness to participate in research. Although examining the influence of healthcare setting was a secondary objective, this aim could only be partially addressed because most studies provided limited information regarding recruitment and participation settings. Consequently, it was not possible to determine whether barriers and facilitators differed meaningfully across primary care, secondary care, community or other healthcare environments.

### Comparison to previous literature

While previous syntheses have identified barriers to research participation among ethnic minority populations, including mistrust, structural inequities, and challenges related to recruitment practices [16, 26], these studies have largely prioritised breadth over specificity. Reviews focusing on women from ethnic minority backgrounds, such as Lovell et al. [31], have typically examined single clinical contexts, limiting transferability across settings. Our findings extend this literature by demonstrating how participation among Black women is shaped by intersecting structural, relational, and cultural influences, and by conceptualising participation as a dynamic process across the research journey. The “bridges and barriers” framework provides an integrated and actionable model to support more inclusive research design.

### Strengths and limitations

Involving PPI representatives at all stages throughout the review process enhanced our understanding of key facilitators and barriers, while ensuring that the findings reflected the lived experiences and perspectives of Black women. Additionally, the inclusion of studies involving Black African and Caribbean women alongside African American women broadened the scope beyond the narrower focus of previous reviews, such as Le et al. [26]. This wider inclusion offers a more comprehensive perspective; however, it also introduces heterogeneity in cultural, historical, and healthcare contexts, which should be acknowledged as a limitation when interpreting the findings. Data extraction and quality appraisal were primarily conducted by a single reviewer, with 20% of studies independently checked by a second reviewer. While this approach reflects accepted pragmatic approaches to systematic reviews, it may have increased the risk of extraction or appraisal errors. However, independent verification identified no substantive discrepancies, providing reassurance regarding the consistency of the review processes. The review challenges simplified assumptions regarding the benefits of racial concordance between researchers and participants. While shared racial or cultural identity was valued by some participants, others expressed concerns about confidentiality, judgement, or tokenism. These findings suggest that racial concordance alone is insufficient and may, in some cases, be counterproductive if not accompanied by genuine cultural competence and reflexive research practices [111, 112]. This highlights the need to move beyond surface-level representation towards more authentic and trust-building forms of engagement.

Despite its strengths, the review highlights significant gaps in the evidence base, particularly the lack of focus on retention strategies and the underrepresentation of studies conducted in the UK and Low- and Middle-Income Countries (LMICs). Although this review explored participation in healthcare research broadly, many included studies examined attitudes towards research in general rather than enrolment into specific clinical trials. As perceptions of burden, risk, and benefit are likely to differ between observational studies and interventional research, including clinical trials, caution is required when interpreting these findings across research contexts. These gaps limit the generalisability and applicability of findings across diverse healthcare contexts. The included papers also did not disaggregate research data by sex and gender. There was variation between the use of the term ‘women’ or ‘female,’ as shown in additional file 4, and as such it was not possible for the review to differentiate results on this basis. Furthermore, the search was restricted to studies published after 2010; while this is a limiting factor, this was imposed given the implementation of the 2010 Equality Act, which marked a shift in policy and practice relevant to the review’s objectives. It should also be noted that whilst the large majority of included papers were based on a participant composition that was 100% Black women, where this was not the case (15 papers, detailed in Additional File 4) it was not possible to extract Black women’s perceptions from other ethnic groups.

While the inclusion of studies from multiple global contexts is a strength of this review, it also introduces variability in healthcare systems, cultural norms, and research infrastructures that may influence how barriers and facilitators are experienced and reported. For example, the dominance of studies from the United States may foreground specific historical drivers of mistrust, whereas in other settings, different sociocultural or system-level factors may shape participation. Likewise, logistical barriers such as access to care, financial burden, and digital inclusion are likely to vary across healthcare contexts. Rather than representing inconsistencies, this variation highlights how common underlying mechanisms (such as trust, accessibility, and relational engagement) manifest differently depending on context. As such, the findings should be interpreted as identifying core patterns that require local adaptation, rather than universally applicable solutions.

### Unanswered questions and future research

Future research should examine how intersectional identities, such as age, disability, and immigration status, shape Black women’s research experiences. With healthcare increasingly delivered digitally, the role of digital positionality [113] also warrants investigation. There is a particular need for UK-based and Low- and Middle-Income Country-focused studies to understand context-specific barriers and facilitators, especially in underrepresented healthcare domains. Co-produced research must move beyond consultation to genuine power-sharing, involving Black women in study design, governance, and dissemination to ensure research is both inclusive and has accountability. This review also highlights a lack of research on retention. Focusing solely on recruitment risks undermining ethical obligations, limits participants’ contributions to primary and secondary outcomes, and reduces the reliability and generalisability of findings. Overall, these insights provide a foundation for developing structured support for research teams to both recruit and retain Black women in healthcare studies.

## Conclusion

This systematic review has provided a comprehensive synthesis of the evidence on barriers and facilitators influencing Black women’s participation in healthcare research. Improving Black women’s participation in healthcare research requires a shift away from narrow recruitment-focused solutions and towards systemic change. By prioritising lived experience and applying a structural, equity-focused framework, this review provides actionable insights for researchers, funders, and institutions seeking to build more inclusive, trustworthy, and effective research systems. The bridges-and-barriers framework offers a novel analytic lens for integrating individual, relational, and structural influences on participation, translating findings into actionable strategies for equitable research. Critically, the review challenges assumptions that symbolic representation or racial concordance alone are sufficient to build trust, these insights call for moving beyond symbolic inclusion towards transparent, accountable, and structurally informed approaches to healthcare research with Black women.

## Supplementary Information

Additional file 1: PRISMA-E 2012 Checklist.

Additional file 2: Search strategy.

Additional file 3: Data extraction tool.

Additional file 4: Characteristics of Included Studies.

Additional file 5: Supporting Evidence for key findings.

Additional file 6: Bridges and Barriers Analogy.

## Data Availability

All data generated by this review are contained in the manuscript and its supplementary files.
