# Supplementary material for: A systematic review and meta synthesis of barriers and facilitators to Black women’s participation in healthcare research
Source: BMC Med Res Methodol. 2026 Jul 21;26:222. doi: 10.1186/s12874-026-02953-7 (PMC13673856; doi:10.1186/s12874-026-02953-7)
Supplement: Supplementary file 2 [file 12874_2026_2953_MOESM2_ESM.docx]

**Additional file 2. Search strategy**

The following search strategy has been identified for **Ovid MEDLINE® ALL** and will be translated for the other databases chosen for this review.

| **#** | **Search term(s)** |
| --- | --- |
| 1 | (("BAME" or BME or "minority ethnic*" or "ethnic minorit*" or "racial* minorit*" or minorit* or "ethnic* group*") adj2 (female* or woman* or women* or girl or girls or mother*)).ti,ab. |
| 2 | (("mixed race" or "mixed racial" or "mixed heritage" or "mixed parentage" or "mixed ethnic*" or "dual heritage" or biracial* or bi-racial* or multiracial* or multi-racial*) adj2 (female* or woman* or women* or girl or girls or mother*)).ti,ab. |
| 3 | ((female* or woman* or women* or girl or girls or mothers*) adj2 (colour or color or black)).ti,ab. |
| 4 | (("black British" or African* or Caribbean*) adj2 (female* or woman* or women* or girl or girls or mother*)).ti,ab. |
| 5 | *Ethnicity/ or *Minority Groups/ or *Minority Health/ or *Black People/ or *Caribbean People/ or *African People/ or *Racial Groups/ or *"Black or African American"/ or *Race Factors/ |
| 6 | exp Women/ |
| 7 | 5 and 6 |
| 8 | 1 or 2 or 3 or 4 or 7 |
| 9 | Patient Participation/ or Patient Selection/ |
| 10 | (retention or retaining or recruit* or participat* or enrol* or include* or participat*).ti,ab. |
| 11 | 9 or 10 |
| 12 | 8 and 11 |
| 13 | ((healthcare or "health care" or clinical or medical or biomedical or hospital or maternal or prenatal or antenatal or maternity or pregnan*) adj2 (research or study or studies or trial*)).ti,ab. |
| 14 | exp Research/ or Biomedical Research/ |
| 15 | 13 or 14 |
| 16 | 12 and 15 |
| 17 | (barrier* or inhibit* or difficulty or difficulties or constrain* or limit* or challeng* or obstacle* or problem* or issue* or interfer* or obstruct* or restrain* or restrict* or disincentive*).tw,kf. |
| 18 | (facilitat* or motivat* or driver* or encourage* or readiness* or success* or enable* or incentive*).tw,kf. |
| 19 | 17 or 18 |
| 20 | 16 and 19 |
| 21 | limit 20 to yr="2010 -Current" |
