# Supplementary material for: A systematic review and meta synthesis of barriers and facilitators to Black women’s participation in healthcare research
Source: BMC Med Res Methodol. 2026 Jul 21;26:222. doi: 10.1186/s12874-026-02953-7 (PMC13673856; doi:10.1186/s12874-026-02953-7)
Supplement: Supplementary file 4 [file 12874_2026_2953_MOESM4_ESM.docx]

**Additional file 4. Characteristics of Included Studies**

| **Author/ Year** | **Country** | **Healthcare topic** | **Sample size (n)** | **Sample ethnicity** | **Sample sex/ gender** | **Study aim** | **Study focusses on:** | | **Study design** |
| --- | --- | --- | --- | --- | --- | --- | --- | --- | --- |
|  |  |  |  |  |  |  | **Recruitment** | **Retention** |  |
| Adams, S. A et al. (2015)^1^ | USA | Oncology | 130 (SISTAS study) | 100% Black | 100% women | To compare and contrast recruitment and retention for a centralized versus a decentralized Community-Based Participatory Research recruitment plan. | Yes | Yes | Quantitative |
| Bachour, C. C et al. (2017)^2^ | USA | OBGYN | 868 | 66% Black,  34% White | 100% women | To compare the effectiveness and efficiency of four recruitment methods and to determine socioeconomic predictors for successful enrolment in a National Institutes of Health–sponsored multicentre clinical trial evaluating a gabapentin intervention in women with provoked vulvodynia. | Yes | No | Quantitative |
| Bardach, S. H et al. (2021)^3^ | USA | Alzheimer's/ Dementia | 21 | 100% Black | 90.5% female | To explore the use of photovoice to add to existing understandings of barriers and facilitators to Alzheimer's disease research participation among African Americans and identify strategies to enhance engagement. | Yes | No | Qualitative |
| Barre, I et al. (2022)^4^ | USA | COVID-19 | 62 | 100% Black | 69.3% female | To apply the theory of planned behaviour to understand motivators and barriers to COVID-19 research participation and how they differ across the study at the initial stages of the COVID-19 pandemic. | Yes | No | Mixed-methods |
| Bass, S. B et al. (2016)^5^ | USA | HIV | 50 | 94% Black,  6% Latino | 56% women | To examine the differences between genders on the perceived barriers and facilitators of being involved in a clinical trial in urban patients of a large HIV clinic that serves predominantly minorities who have never participated in clinical research. | Yes | No | Quantitative |
| Berg, K. A et al. (2025)^6^ | USA | Sleep apnoea | 18 | 100% Black | 60% female | To determine the perceptions of African American patients with obstructive sleep apnoea and their bed partners regarding participating in clinical trials evaluating the effects of CPAP on long-term outcomes. | Yes | No | Qualitative |
| Blanchet, R et al. (2017)^7^ | Canada | Child nutrition | 259 | 81.4% Black,  15.1% white,  3.5% other/mixed | 98.8% women | To offer insights on strategies and challenges in recruiting black immigrant mothers living in Ottawa (Canada) for a community-based health-oriented research project among 6-to-12-year-old children. | Yes | No | Mixed-methods |
| Bontemps-Jones, J. E et al. (2024)^8^ | USA | Research | 38 | 100% Black | 100% women | To better understand and contextualize the contemporary barriers to research participation among Black women and guide subsequent research to be more inclusive. | Yes | No | Qualitative |
| Brewer, L. C et al (2021)^9^ | USA | Research | 29 | 90% Black,  5% white,  5% DNA | 71% women | To investigate best practices and strategies for enhancing participation of African Americans in medical research among attendees of a preconference Institute at a faith based public health conference. | Yes | No | Qualitative |
| Brown, R. F et al. (2013)^10^ | USA | Oncology | 22 | 100% Black | 68.2% female | To (1) explore trial refusal reasons in a sample of African American patients with cancer who declined trial participation and (2) gather patients’ perceptions of the potential beneﬁt of an array of decision support tools. | Yes | No | Mixed-methods |
| Buseh, A. G et al. (2013)^11^ | USA | Genetics | 21 | 100% Black | 81% female | To: (a) explore African Americans’ perspectives on genetics/genomic research, (b) understand facilitators and barriers to participation in such studies, and (c) enlist their ideas about how to attract and sustain engagement of African Americans in genetics initiatives. | Yes | No | Qualitative |
| Canidate, S. S et al. (2020)^12^ | USA | HIV and alcohol use | 20 | 85% Black,  15% white,  5% other | 100% women | To better understand the reasons why women with HIV and hazardous drinking participated in the WHAT-IF? study and to discuss their experience (positive or negative) in the study. | Yes | Yes | Qualitative |
| Castillo, A. G et al. (2012)^13^ | USA | Oncology | 115 | 100% Black | 73.9% female | To understand the benefits participants reported experiencing as a result of their participation in a research study. | Yes | Yes | Qualitative |
| Chung, J. E et al (2025)^14^ | USA | Research | 28 | 64% Black,  36% Latino | 75% women | To understand what Black and Latino community members know about clinical trials and develop effective messaging to generate interest, improve access, and encourage participation among minoritized populations through social media. | Yes | No | Qualitative |
| Colon-Otero, G et al. (2012)^15^ | USA | Oncology | 318 | 77% Black,  19% white,  2% Asian,  2% Hispanic  /Latino | 72% female | To evaluate whether collaboration with African American churches can improve cancer awareness and increase participation in translational research protocols among African Americans. | Yes | No | Quantitative |
| Cox, L. S et al. (2011)^16^ | USA | Smoking cessation | 540 | 100% Black | 66% women | To describe the study design, baseline characteristics and retention of African American light smokers into a smoking cessation treatment trial that involves biological data collection. | Yes | Yes | Quantitative |
| Cudjoe, J et al. (2019)^17^ | USA | Oncology | 167 | 100% Black | 100% women | To describe experiences and lessons learned in recruiting African immigrant women for the AfroPap study, a community-based study examining correlates of cervical cancer screening behaviours. | Yes | No | Mixed-methods |
| Davis, K et al (2025)^18^ | USA | Covid-19 | 236 | 100% Black | 78.4% female | To use the health belief model to explore access to and use of Covid-19 preventative materials, preventative health behaviours, and willingness to participate in research during the Covid-19 pandemic in older African Americans. | Yes | No | Quantitative |
| Disbrow, E. A et al. (2021)^19^ | USA | Alzheimer's/ Dementia | 59 | 78% Black,  22% white | 92% female | To identify barriers to resource access and use, as well as to develop health information recommendations for caregivers, clinicians, and researchers. | Yes | No | Mixed-methods |
| Dobreva, I et al. (2025)^20^ | UK | Parkinson’s disease | 17 | 100% Black | 64.7% female | To explore the factors affecting participation in Parkinson’s disease research in Black populations living in UK. | Yes | No | Qualitative |
| Duhaney. P (2024)^21^ | Canada | Intimate partner violence | 25 | 100% Black | 100% women | To examine Canadian Black women’s motivations for participating in the research study that explored their experiences with the police in the context of intimate partner violence and the key factors that complicated their decisions. | Yes | No | Qualitative |
| Eliacin, J et al. (2022)^22^ | USA | Alzheimer's/ Dementia | 32 | 57.7% Black,  38.5% white,  3.8% multiple ethnicity | 57.7% female | To examine participants’ perceived barriers to study participation and drivers of engagement in Alzheimer's disease biomarker research, while seeking to identify differences between Black and White participants. | Yes | Yes | Qualitative |
| Evans, K et al. (2012)^23^ | USA | Oncology | 50 | 58% Black,  42% Hispanic /Latino | 77.2% female | To explore African Americans’ and Hispanics’ perceptions about cancer clinical trials using Zarcadoolas, et al. multidimensional health literacy framework as a conceptual guide. | Yes | No | Qualitative |
| Fairley, R et al. (2024)^24^ | USA | Oncology | 305 | 100% Black | 99.8% female | To engage Black women to examine clinical trial awareness and perceptions; to identify various cultural, emotional, and social barriers and motivators to participation; and to identify and quantify levels of mistrust in medical research. | Yes | No | Mixed-methods |
| Fincham, D et al. (2010)^25^ | South Africa | HIV | 264 | 100% Black | 100% female | To identify and validate with a larger sample the factors that may inhibit or facilitate willingness to participate in an HIV vaccine trial among high risk South Africans. | Yes | No | Quantitative |
| Fouad, M. N et al. (2016)^26^ | USA | Oncology | 272 | 100% Black | 71% female | To report on the impact of the Increasing Minority Participation in Clinical Trials (IMPaCT) project using patient navigation over a period of 8 years. | Yes | Yes | Quantitative |
| Frederick, H et al. (2022)^27^ | USA | Sexual health | 39 | 100% Black | 100% women | To understand Black women’s openness to participating in sexual health related research. | Yes | No | Qualitative |
| Frew, P. M et al. (2010)^28^ | USA | HIV | 362 | 100% Black | 63.3% female | To test a model of reasoned action that assesses not only the interplay of attitudes, beliefs, and normative concerns, but includes perceptions of the clinical research entity sponsoring vaccine trials as well as perceptions of social activism, in formulating decisions to participate in the search for a vaccine. | Yes | No | Quantitative |
| Frierson, G. M et al. (2019)^29^ | USA | Oncology | 57 | 100% Black | 100% women | To identify research perceptions and motivating factors needed to increase racial/ethnic minority participation in breast cancer research. | Yes | No | Mixed-methods |
| Gatny, H and Axinn, W. G. (2012)^30^ | USA | OBGYN | 90 | 54% Black,  46% white | 100% women | To examine the willingness of pregnant women to participate in research on health. | Yes | No | Mixed-methods |
| Gebremedhen, T. et al. (2025)^31^ | Canada | HIV | 56 | 100% Black | 78.6% women | To understand research experiences and priorities among African, Caribbean and Black people in British Columbia. | Yes | No | Quantitative |
| Granda-Cameron, C et al. (2022)^32^ | USA | Oncology | 13 | 100% Black | 92.3% female | To explore the perceptions of African American survivors and caregivers about participation in clinical trials. | Yes | No | Qualitative |
| Gunn, A. J et al. (2021)^33^ | USA | Substance abuse | 28 | 100% Black | 100% women | To explore the perceptions of African American survivors and caregivers about participation in clinical trials. | Yes | No | Qualitative |
| Gwizdala, K. L et al. (2022)^34^ | USA | COVID-19 | 624 | 100% Black | 54% female | To assess the impact of COVID-19 on barriers and willingness to participate in research among older African Americans. | Yes | No | Mixed-methods |
| Haynes-Maslow, L et al. (2014)^35^ | USA | Oncology | 82 | 100% Black | 100% women | To determine the perceived barriers and facilitators to participating in cancer clinical trials. | Yes | Yes | Mixed-methods |
| Hughes, T. B et al. (2017)^36^ | USA | Research | 64 | 96.9% Black,  3.1% white | 71.9% female | To examine barriers and facilitators to clinical research participation among African Americans, as well as recommendations for overcoming these. | Yes | No | Mixed-methods |
| James. D. C (2016)^37^ | USA | E-health | 589 | 100% Black | 100% women | To (1) examine African American women’s use of and access to technology, (2) identify motivators and barriers to participating in e-health/m-health research, and (3) determine if African American women’s use, access, motivators, and barriers varied by age group. | Yes | No | Quantitative |
| Johnson, C. K et al. (2024)^38^ | USA | Oncology | 807 | 100% Black | 52.5% female | To examine effective recruitment strategies in Black communities of New York City that were utilized during data collection for part of a larger study examining colorectal cancer screening behaviours and intent. | Yes | No | Quantitative |
| Kapiriri, L et al. (2017)^39^ | Canada | HIV | 27 | 100% Black | 100% women | To (1) describe the cultural beliefs about the uses of breast milk/fluids, among HIV+ African, Caribbean and Black women in Hamilton and Toronto Ontario, Canada, (2) establish the acceptability of using breast milk/fluids for research purposes, and (3) determine the culturally acceptable approaches to collecting breast milk/fluids and other biological samples from these populations. | Yes | No | Qualitative |
| Lang, R. et al. (2013)^40^ | USA | Research | 733 | 100% Black | 60% women | To elucidate factors that influence African American willingness to participate in health-related research studies. | Yes | No | Quantitative |
| Lincoln, K. D et al. (2021)^41^ | USA | Alzheimer's/ Dementia | 44 | 100% Black | 70.4% women | To examine the roles of race and culture in the attitudes and beliefs of African Americans about participating in clinical research. | Yes | No | Qualitative |
| Malova, E. et al. (2025)^42^ | USA | Research | 48 | 100% Black | 61% female | To examine how African American and Black Caribbean patients articulate motivations and concerns about clinical trial participation, and how clinical research coordinators perceive and respond to these beliefs during recruitment. | Yes | No | Qualitative |
| Morgan, S. E et al. (2023)^43^ | USA | Research | 49 | 100% Black | 61.2% female | To examine the role of expertise and trustworthiness in shaping the attitudes of prospective research participants by analysing data gathered from both clinical research coordinators and Black/African American patients. | Yes | No | Qualitative |
| Morgan, S. E et al. (2024)^44^ | USA | Research | 31 | 100% Black | 61.2% female | To focuses on the communication factors that affect the willingness of African Americans and Black Caribbean patients to participate in clinical trials and research studies. | Yes | No | Qualitative |
| Newton, V. et al. (2025)^45^ | USA | Covid-19 | 52 | 100% Black | 50% women | To better understand the barriers and motivations for COVID-19 testing among Black Americans | Yes | No | Qualitative |
| Ngui, E. M et al. (2014)^46^ | USA | Genetics | 46 | 100% Black | 80% women | To examine the perspectives of African-American health professional and community members on genetic research involving children and pregnant women. | Yes | No | Mixed-methods |
| O'Connor, M et al. (2018)^47^ | USA | Research | 22 | 100% Black | 77% female | To (a) investigate young African Americans’ knowledge about and attitudes toward health registries and (b) explore their perceptions about current messages used to recruit participants into health registries. | Yes | No | Qualitative |
| Occa, A et al. (2023)^48^ | USA | Oncology | 20 | 100% Black | 75% female | To try to illuminate how providers can better engage Black women in communication that will positively influence their perceptions of breast cancer clinical trial participation. | Yes | No | Mixed-methods |
| Okoruwa, P and Ridley-Merriweather, K. E (2022)^49^ | USA | Paediatrics | 14 | 100% Black | 100% women | To gain a greater understanding of perceptions related to barriers/facilitators for paediatric clinical research participation among East African immigrant community members. | Yes | No | Qualitative |
| Olakunde, B. et al. (2025)^50^ | Nigeria | Oncology | 60 | 100% Black | 54.3% female | To assess health care workers’ perspectives on recruitment and retention in cancer clinical trials in Nigeria. | Yes | Yes | Mixed-methods |
| Omondi, N. A et al. (2013)^51^ | USA | Sickle cell disease | 51 | 100% Black | 74.5% female | To better understand participation barriers to hematopoietic cell transplantation clinical trials for sickle-cell disease. | Yes | No | Qualitative |
| Overstreet, N. M and Cheeseborough, T (2020)^52^ | USA | HIV | 87 | 100% Black | 100% women | To examine African American women’s experiences of internalized HIV-related stigma and how these experiences shape their perceptions of research participation. | Yes | No | Quantitative |
| Overstreet, N. M et al. (2018)^53^ | USA | HIV and Intimate partner violence | 89 | 100% Black | 100% women | To capture African American women’s attitudes toward their participation in a study focusing on their HIV status and intimate partner violence experiences. | Yes | No | Mixed-methods |
| Poteat, T et al. (2020)^54^ | USA | HIV | 10 | 100% Black | 100% transgender women | To address the gap in knowledge by eliciting Black transgender women’s opinions of HIV cure-related research and factors that may affect their willingness to take part in these studies. | Yes | No | Qualitative |
| Princewell, C. W et al. (2017)^55^ | Nigeria | Research | 54 | 100% Black | ~74% women | To explore the experience and understanding of autonomy by the Yoruba women in relation to research participation. | Yes | No | Qualitative |
| Pugh, E. et al. (2022)^56^ | USA | Alzheimer's/ Dementia | 51 | 100% Black | 76.5% female | The purpose of the study was to collect data related to beliefs toward dementia in older African Americans residing in Southern USA. | Yes | No | Qualitative |
| Reddy, A et al. (2020)^57^ | USA | Biospecimen donation | 294 | 85.8% Black,  14.2% other ethnicities | 71.9% female | To define the psychosocial factors contributing to current disparity in biospecimen collection for research studies. | Yes | No | Quantitative |
| Ridley-Merriweather, K. et al. (2024)^58^ | USA | Oncology | 14 | 100% Black | 100% women | To investigate patient-provider conversations to try to illuminate how providers can better engage Black women in communication that will positively influence their perceptions of breast cancer clinical trial participation. | Yes | No | Qualitative |
| Ridley-Merriweather, K. et al. (2025)^59^ | USA | Oncology | 14 | 100% Black | 100% women | To better understand what may motivate Black women to engage in medical research and decide to participate in medical research. | Yes | No | Qualitative |
| Rivers, D. A et al. (2019)^60^ | USA | Oncology | 42 | 100% Black | 100% women | To explore informational needs regarding cancer clinical trials among African American women and identify message considerations for educational information targeting African American women and their community. | Yes | No | Qualitative |
| Rollins, L et al. (2018)^61^ | USA | Research | 60 | 100% Black | 53.3% female | To increase African Americans’ knowledge about clinical research and willingness to participate in clinical research and improve their perceptions and attitudes about clinical research. | Yes | No | Mixed-methods |
| Scharf, D. P et al. (2010)^62^ | USA | Oncology and Alzheimer's/ Dementia | 70 | 100% Black | 72.6% female | To identify barriers to research participation, including more complex studies, and then use the findings to develop interventions to improve participation in both cancer and Alzheimer’s-related trials. | Yes | No | Qualitative |
| Shaia, W. E et al. (2020)^63^ | USA | Autism | 22 | 90.8% Black | 100% women | To explore facilitators and barriers to research participation; as well as suggestions to increase their involvement in autism spectrum disorder studies. | Yes | No | Qualitative |
| Shiyanbola, O. O et al. (2025)^64^ | USA | Research | 14 | 100% Black | 57% female | To investigate the use of an adapted methods-motivational interviewing approach in enrolling Black adults into a trial, investigating their facilitators and barriers to trial enrolment. | Yes | Yes | Qualitative |
| Smith, A et al. (2018)^65^ | USA | Oncology | 364 | 100% Black | 100% women | The objective of this study was to evaluate the feasibility of utilizing a community-based participatory research approach for recruiting and retaining African American women in Memphis in the STAR study. | Yes | No | Quantitative |
| Sneed, R. S et al. (2021)^66^ | USA | Lupus | 31 | 100% Black | 90% female | To explore the influence of racism on the underrepresentation of Black patients in systemic lupus erythematous clinical trials and to develop a framework for future intervention. | Yes | No | Qualitative |
| Somayaji, D and Cloyes, K. G (2015)^67^ | USA | Oncology | 16 | 100% Black | 62.5% women | To explore the perceptions of African Americans regarding cancer and research and how these perceptions shape their beliefs about participating as cancer research subjects. | Yes | No | Qualitative |
| Strekalova, Y. A (2018)^68^ | USA | Research | 340 | 50% Black,  50% white | 50% female | To explore the effect of race identity, e-health information efficacy, and information behaviour on intention to participate in clinical research. | Yes | No | Quantitative |
| Voytek, C. D et al. (2011)^69^ | USA | HIV | 17 | 100% Black | 100% female | To examine factors associated with participation in an actual HIV vaccine trial among African-American women in Philadelphia. | Yes | No | Qualitative |
| White, D. B et al. (2010)^70^ | USA | Oncology | 67 | 100% Black | 70% female | To assess whether reactions to genetic explanations for disparities in lung cancer incidence among family members of African American patients with lung cancer are associated with willingness to participate in clinical genetics research. | Yes | No | Quantitative |
| Williams, M. M et al. (2010)^71^ | USA | Alzheimer's/ Dementia | 70 | 100% Black | 73% female | To examine barriers and facilitators of Alzheimer’s disease research participation among African Americans. | Yes | No | Qualitative |
| Williams, M.T et al. (2013)^72^ | USA | Mental health | 6 | 100% Black | 60% female | To examine why African Americans might be reluctant to participate in anxiety disorder studies. | Yes | No | Qualitative |

1.	Adams SA, Heiney SP, Brandt HM, et al. A comparison of a centralized versus de-centralized recruitment schema in two community-based participatory research studies for cancer prevention. *Journal of community health* 2015; 40: 251-259. DOI: doi:<https://dx.doi.org/10.1007/s10900-014-9924-9>.

2.	Bachour CC, Bachmann GA, Foster DC, et al. Recruitment methods in a clinical trial of provoked vulvodynia: Predictors of enrollment. *Clinical Trials* 2017; 14: 103-108. DOI: doi:<https://dx.doi.org/10.1177/1740774516663461>.

3.	Bardach SH, Yarbrough M, Walker C, et al. Insights From African American Older Adults on Brain Health Research Engagement: "Need to See the Need". *Journal of Applied Gerontology* 2021; 40: 201-208. DOI: doi:<https://dx.doi.org/10.1177/0733464820902002>.

4.	Barre I, Cunningham-Erves J, Moss J, et al. Motivators and Barriers to COVID-19 Research Participation at the Onset of the COVID-19 Pandemic in Black Communities in the USA: an Exploratory Study. *Journal of Racial & Ethnic Health Disparities* 2023; 10: 2890-2899. DOI: doi:10.1007/s40615-022-01466-5.

5.	Bass SB, Wolak C, Greener J, et al. Using perceptual mapping methods to understand gender differences in perceived barriers and benefits of clinical research participation in urban minority HIV+ patients. *AIDS Care* 2016; 28: 528-536. DOI: doi:10.1080/09540121.2015.1112352.

6.	Berg KA, Marbury M, Whaley MA, et al. Perspectives of African American couples regarding participation in trials of treatments for obstructive sleep apnea. *Journal of the National Medical Association* 2025; 117: 449-457. DOI: <https://doi.org/10.1016/j.jnma.2025.08.106>.

7.	Blanchet R, Sanou D, Nana CP, et al. Strategies and Challenges in Recruiting Black Immigrant Mothers for a Community-Based Study on Child Nutritional Health in Ottawa, Canada. *Journal of Immigrant & Minority Health* 2017; 19: 367-372. DOI: doi:<https://dx.doi.org/10.1007/s10903-016-0536-6>.

8.	Bontemps-Jones JE, McCullough LE, Kirkland EG, et al. Beyond Tuskegee: A contemporary qualitative assessment of barriers to research participation among Black women. *Cancer* 2025; 131: e35648. DOI: <https://doi.org/10.1002/cncr.35648>.

9.	Brewer LPC, Pasha M, Seele P, et al. Overcoming Historical Barriers: Enhancing Positive Perceptions of Medical Research Among African Americans Through a Conference-Based Workshop. *Journal of General Internal Medicine* 2021; 36: 2547-2554. DOI: doi:<https://dx.doi.org/10.1007/s11606-021-06736-2>.

10.	Brown RF, Cadet DL, Houlihan RH, et al. Perceptions of Participation in a Phase I, II, or III Clinical Trial Among African American Patients With Cancer: What Do Refusers Say? *Journal of Oncology Practice* 2013; 9: 287-293. DOI: doi:10.1200/JOP.2013.001039.

11.	Buseh AG, Stevens PE, Millon-Underwood S, et al. Community leaders' perspectives on engaging African Americans in biobanks and other human genetics initiatives. *Journal of Community Genetics* 2013; 4: 483-494. DOI: doi:<https://dx.doi.org/10.1007/s12687-013-0155-z>.

12.	Canidate SS, Cook CL, Varma D, et al. Recruitment, experience, and retention among women with HIV and hazardous drinking participating in a clinical trial. *BMC Public Health* 2020; 20: 1169. DOI: doi:<https://dx.doi.org/10.1186/s12889-020-09233-z>.

13.	Castillo AG, Jandorf L, Thelemaque LD, et al. Reported benefits of participation in a research study. *Journal of community health* 2012; 37: 59-64. DOI: doi:.

14.	Chung JE, Cramer EM, Uche S, et al. Engaging Minoritized Communities in Clinical Trials Through Social Media: Recommendations from Community-Based Participatory Research. *Health Communication* 2025; 40: 2696-2704. DOI: 10.1080/10410236.2025.2476684.

15.	Colon-Otero G, Albertie M, Lesperance M, et al. A pilot program in collaboration with African American churches successfully increases awareness of the importance of cancer research and participation in cancer translational research studies among African Americans. *Journal of Cancer Education* 2012; 27: 294-298. DOI: doi:10.1007/s13187-011-0288-x.

16.	Cox LS, Faseru B, Mayo MS, et al. Design, baseline characteristics, and retention of African American light smokers into a randomized trial involving biological data. *Trials* 2011; 12: 22-22. DOI: doi:10.1186/1745-6215-12-22.

17.	Cudjoe J, Turkson-Ocran R-A, Ezeigwe AK, et al. Recruiting African Immigrant Women for Community-Based Cancer Prevention Studies: Lessons Learned from the AfroPap Study. *Journal of Community Health* 2019; 44: 1019-1026. DOI: doi:10.1007/s10900-019-00677-y.

18.	Davis K, Pugh E, Beyl R, et al. Responses to Covid-19 and Research Participation Among Older African Americans. *Journal of Applied Gerontology*; 0: 07334648251369670. DOI: 10.1177/07334648251369670.

19.	Disbrow EA, Arnold CL, Glassy N, et al. Alzheimer Disease and Related Dementia Resources: Perspectives of African American and Caucasian Family Caregivers in Northwest Louisiana. *Journal of Applied Gerontology* 2021; 40: 209-219. DOI: doi:10.1177/0733464820904568.

20.	Dobreva I, Kalam S, Roche M, et al. Understanding barriers and facilitators to participation in Parkinson's research in Black communities in the UK. *Journal of Parkinson’s Disease* 2025; 15: 1035-1039. DOI: 10.1177/1877718x251351990.

21.	Duhaney P. Fostering Change: Black Women's Motivations for Participating in Intimate Partner Violence Research. *Affilia: Feminist Inquiry in Social Work* 2024; 39: 265-284. DOI: doi:10.1177/08861099231187861.

22.	Eliacin J, Hathaway E, Wang S, et al. Factors influencing the participation of Black and White Americans in Alzheimer's disease biomarker research. *Alzheimer's and Dementia: Diagnosis, Assessment and Disease Monitoring* 2022; 14. DOI: doi:<https://dx.doi.org/10.1002/dad2.12384>.

23.	Evans K, Lewis M, Hudson S, et al. The role of health literacy on African American and Hispanic/Latino perspectives on cancer clinical trials. *Journal of Cancer Education* 2012; 27: 299-305. DOI: doi:10.1007/s13187-011-0300-5.

24.	Fairley R, Lillard JW, Jr., Berk A, et al. Increasing Clinical Trial Participation of Black Women Diagnosed with Breast Cancer. *Journal of Racial & Ethnic Health Disparities* 2024; 11: 1701-1717. DOI: doi:<https://dx.doi.org/10.1007/s40615-023-01644-z>.

25.	Fincham D, Kagee A and Swartz L. Inhibitors and facilitators of willingness to participate (WTP) in an HIV vaccine trial: Construction and initial validation of the Inhibitors and Facilitators of Willingness to Participate Scale (WPS) among women at risk for HIV infection. *AIDS Care - Psychological and Socio-Medical Aspects of AIDS/HIV* 2010; 22: 452-461. DOI: doi:<https://dx.doi.org/10.1080/09540120903202939>.

26.	Fouad MN, Acemgil A, Bae S, et al. Patient Navigation As a Model to Increase Participation of African Americans in Cancer Clinical Trials. *Journal of Oncology Practice* 2016; 12: 554-565. DOI: doi:10.1200/JOP.2015.008946.

27.	Frederick H, Wade J, Parker S, et al. Understanding Openness to Involvement in Sexual Health Care Research: Narratives from a Sample of Black College Women in the United States. *Journal of Sex Research* 2022; 59: 1122-1132. DOI: doi:<https://dx.doi.org/10.1080/00224499.2021.1994515>.

28.	Frew PM, Archibald M, Diallo DD, et al. An extended model of reasoned action to understand the influence of individual- and network-level factors on African Americans' participation in hiv vaccine research. *Prevention Science* 2010; 11: 207-218. DOI: doi:<https://dx.doi.org/10.1007/s11121-009-0162-9>.

29.	Frierson GM, Pinto BM, Denman DC, et al. Bridging the Gap: Racial concordance as a strategy to increase African American participation in breast cancer research. *Journal of Health Psychology* 2019; 24: 1548-1561. DOI: doi:<https://dx.doi.org/10.1177/1359105317740736>.

30.	Gatny HH and Axinn WG. Willingness to participate in research during pregnancy: Race, experience, and motivation. *Field Methods* 2012; 24: 135-154. DOI: doi:<https://dx.doi.org/10.1177/1525822X11419819>.

31.	Gebremedhen T, Campbell AR, Magagula P, et al. Understanding Research Participation Experiences Among Persons Identifying as African, Caribbean, and Black in British Columbia. *Prog Community Health Partnersh* 2025; 19: 169-178. DOI: 10.1353/cpr.2025.a965354.

32.	Granda-Cameron C, McLean Florence Y, Whitfield-Harris L, et al. Perceptions of Clinical Trial Participation in African American Cancer Survivors and Caregivers. *Oncology Nursing Forum* 2022; 49: 113-124. DOI: doi:10.1188/22.ONF.113-124.

33.	Gunn AJ, Hardesty M, Overstreet N, et al. "Every time I tell my story I learn something new": Voice and inclusion in research with Black women with histories of substance use and incarceration. *Criminology & Criminal Justice: An International Journal* 2022; 22: 505-524. DOI: doi:<https://dx.doi.org/10.1177/17488958211005820>.

34.	Gwizdala KL, Pugh EA, Carter L, et al. Impact of COVID-19 Pandemic on Research Participation among Older African Americans. *Alzheimer Disease and Associated Disorders* 2022; 36: 350-353. DOI: doi:<https://dx.doi.org/10.1097/WAD.0000000000000512>.

35.	Haynes-Maslow L, Godley P, Dimartino L, et al. African American women's perceptions of cancer clinical trials. *Cancer Medicine* 2014; 3: 1430-1439. DOI: doi:<https://dx.doi.org/10.1002/cam4.284>.

36.	Hughes TB, Varma VR, Pettigrew C, et al. African Americans and Clinical Research: Evidence Concerning Barriers and Facilitators to Participation and Recruitment Recommendations. *The Gerontologist* 2017; 57: 348-358. DOI: doi:<https://dx.doi.org/10.1093/geront/gnv118>.

37.	James DC, Harville C, 2nd, Whitehead N, et al. Willingness of African American Women to Participate in e-Health/m-Health Research. *Telemedicine Journal & E-Health* 2016; 22: 191-197. DOI: doi:<https://dx.doi.org/10.1089/tmj.2015.0071>.

38.	Johnson CK, Leung MM, Ma GX, et al. Effective Recruitment Strategies Utilized to Examine Dietary Practices of Blacks in New York City in the Midst of the COVID-19 Pandemic. *Journal of Racial & Ethnic Health Disparities* 2024; 11: 764-772. DOI: doi:10.1007/s40615-023-01559-9.

39.	Kapiriri L, Tharao W, Muchenje M, et al. How acceptable is it for HIV positive African, Caribbean and Black women to provide breast milk/fluid samples for research purposes? *BMC Research Notes* 2017; 10: 7. DOI: doi:<https://dx.doi.org/10.1186/s13104-016-2326-6>.

40.	Lang R, Kelkar VA, Byrd JR, et al. African American participation in health-related research studies: indicators for effective recruitment. *Journal of public health management and practice : JPHMP* 2013; 19: 110-118. DOI: doi:.

41.	Lincoln KD, Chow T, Gaines BF, et al. Fundamental causes of barriers to participation in Alzheimer's clinical research among African Americans. *Ethnicity & health* 2021; 26: 585-599. DOI: doi:<https://dx.doi.org/10.1080/13557858.2018.1539222>.

42.	Malova E, Jia X, Harrison TR, et al. Tensions Within Incentives, Altruism, and Trust in Clinical Trial Participation: Perspectives From African American and Black Caribbean Patients and Clinical Research Coordinators Who Recruit Them. *Journal of Racial and Ethnic Health Disparities* 2025. DOI: 10.1007/s40615-025-02701-5.

43.	Morgan SE, Harrison TR, Wright KO, et al. The role of perceived expertise and trustworthiness in research study and clinical trial recruitment: Perspectives of clinical research coordinators and African American and Black Caribbean patients. *PLOS One* 2023; 18. DOI: doi:<https://dx.doi.org/10.1371/journal.pone.0275770>.

44.	Morgan SE, Harrison TR, Wright KO, et al. Reducing Health Disparities Among African American and Black Caribbean Patients by Improving the Communication Practices of Clinical Research Coordinators. *Health communication* 2024; 39: 1298-1309. DOI: doi:<https://dx.doi.org/10.1080/10410236.2023.2211740>.

45.	Newton V, Farinu O, Smith H, et al. Speaking Out: Factors Influencing Black Americans’ Engagement in COVID-19 Testing and Research. *Journal of Racial and Ethnic Health Disparities* 2025. DOI: 10.1007/s40615-024-02268-7.

46.	Ngui EM, Warner TD and Roberts LW. Perceptions of African-American health professionals and community members on the participation of children and pregnant women in genetic research. *Public Health Genomics* 2014; 17: 23-32. DOI: doi:<https://dx.doi.org/10.1159/000355359>.

47.	O'Connor MR, Adem A and Starks H. East African Perceptions of Barriers/Facilitators for Pediatric Clinical Research Participation and Development of the Inclusive Research Model. *Journal of Pediatric Nursing* 2018; 42: 104-110. DOI: doi:10.1016/j.pedn.2018.05.005.

48.	Occa A, Francis DB, Grumbein A, et al. Improving African Americans' Intentions to Enroll in a Health Registry: Message Development and Evaluation. *Health Communication* 2023; 38: 2884-2893. DOI: doi:10.1080/10410236.2022.2124061.

49.	Okoruwa P and Ridley-Merriweather KE. Examining recollections of Black women with breast cancer who participated in clinical trials: A grounded practical theory study of patient-provider communication. *Cancer Research Conference* 2022; 83. DOI: doi:<https://dx.doi.org/10.1158/1538-7445.SABCS22-P6-09-03>.

50.	Olakunde BO, Idemili-Aronu N, Friebel-Klingner TM, et al. Optimizing Recruitment and Retention in Cancer Clinical Trials in Low-Resource Settings: Barriers and Facilitators From Nigerian Provider's Perspectives. *JCO Glob Oncol* 2025; 11: e2400308. 20250213. DOI: 10.1200/go-24-00308.

51.	Omondi NA, Ferguson SES, Majhail NS, et al. Barriers to hematopoietic cell transplantation clinical trial participation of African American and black youth with sickle cell disease and their parents. *Journal of Pediatric Hematology/Oncology* 2013; 35: 289-298. DOI: doi:<https://dx.doi.org/10.1097/MPH.0b013e31828d5e6a>.

52.	Overstreet NM and Cheeseborough T. Examining the Effect of Internalized HIV-Related Stigma on Perceptions of Research Participation among HIV-Positive African American Women. *Translational Issues in Psychological Science* 2020; 6: 223-234. DOI: doi:<https://dx.doi.org/10.1037/tps0000271>.

53.	Overstreet NM, Okuyan M and Fisher CB. Perceived Risks and Benefits in IPV and HIV Research: Listening to the Voices of HIV-Positive African American Women. *Journal of Empirical Research on Human Research Ethics* 2018; 13: 511-524. DOI: doi:<https://dx.doi.org/10.1177/1556264618797557>.

54.	Poteat T, Aqil A, Corbett D, et al. "I would really want to know that they had my back": Transgender women's perceptions of HIV cure-related research in the United States. *PLoS ONE [Electronic Resource]* 2020; 15: e0244490. DOI: doi:<https://dx.doi.org/10.1371/journal.pone.0244490>.

55.	Princewill CW, Jegede AS, Nordström K, et al. Factors Affecting Women's Autonomous Decision Making In Research Participation Amongst Yoruba Women Of Western Nigeria. *Developing World Bioethics* 2017; 17: 40-49. DOI: doi:10.1111/dewb.12112.

56.	Pugh E, Stewart J, Carter L, et al. Beliefs, Understanding, and Barriers Related to Dementia Research Participation Among Older African Americans. *Alzheimer Disease & Associated Disorders* 2022; 36: 52-57. DOI: doi:10.1097/WAD.0000000000000476.

57.	Reddy A, Amarnani A, Chen M, et al. Privacy Concerns About Personal Health Information and Fear of Unintended Use of Biospecimens Impact Donations by African American Patients. *Journal of Cancer Education* 2020; 35: 522-529. DOI: doi:10.1007/s13187-019-01491-9.

58.	Ridley-Merriweather KE, Okoruwa OP and Vogel K. Examining recollections of Black women with breast cancer who participated in clinical trials: A grounded practical theory study of patient-provider communication. *Contemporary Clinical Trials Communications* 2024; 42: 101403. DOI: <https://doi.org/10.1016/j.conctc.2024.101403>.

59.	Ridley-Merriweather KE, Head KJ, Brann M, et al. “We Don’t Get Drugs Targeted for Us:” Applying the Integrated Behavioral Model to Understand Why Black Women Chose to Participate in a Breast Cancer Clinical Trial. *Health Communication* 2025; 40: 2280-2289. DOI: 10.1080/10410236.2024.2448701.

60.	Rivers DA, Pal T, Vadaparampil ST, et al. A community-academic partnership to explore informational needs of African American women as a primer for cancer clinical trial recruitment. *Ethnicity & Health* 2019; 24: 679-693. DOI: doi:<https://dx.doi.org/10.1080/13557858.2017.1367762>.

61.	Rollins L, Sy A, Crowell N, et al. Learning and action in community health: Using the health belief model to assess and educate African American community residents about participation in clinical research. *International Journal of Environmental Research and Public Health* 2018; 15. DOI: doi:<https://dx.doi.org/10.3390/ijerph15091862>.

62.	Scharf DP, Mathews KJ, Jackson P, et al. More than Tuskegee: Understanding mistrust about research participation. *Journal of Health Care for the Poor and Underserved* 2010; 21: 879-897. DOI: doi:<https://dx.doi.org/10.1353/hpu.0.0323>.

63.	Shaia WE, Nichols HM, Dababnah S, et al. Brief Report: Participation of Black and African-American Families in Autism Research. *Journal of Autism and Developmental Disorders* 2020; 50: 1841-1846. DOI: doi:<https://dx.doi.org/10.1007/s10803-019-03926-0>.

64.	Shiyanbola OO, Wen M-J and Maurer MA. Incorporating health literacy principles into the adaptation of a methods motivational interviewing approach for enrolling black adults in a pilot randomized trial. *Scientific Reports* 2025; 15: 4833. DOI: 10.1038/s41598-025-89413-2.

65.	Smith A, Vidal GA, Pritchard E, et al. Sistas Taking a Stand for Breast Cancer Research (STAR) Study: A Community-Based Participatory Genetic Research Study to Enhance Participation and Breast Cancer Equity among African American Women in Memphis, TN. *International Journal of Environmental Research & Public Health [Electronic Resource]* 2018; 15: 18. DOI: doi:<https://dx.doi.org/10.3390/ijerph15122899>.

66.	Sneed RS, Mason M, Williams JN, et al. Using Critical Race Theory to Understand Trial Participation Among Black Individuals With Systemic Lupus Erythematosus: A Qualitative Study of Patients and Caregivers. *Arthritis Care and Research* 2021; 73: 1387-1395. DOI: doi:<https://dx.doi.org/10.1002/acr.24635>.

67.	Somayaji D and Cloyes KG. Cancer fear and fatalism: How African American participants construct the role of research subject in relation to clinical cancer research. *Cancer Nursing* 2015; 38: 133-144. DOI: doi:<https://dx.doi.org/10.1097/NCC.0000000000000144>.

68.	Strekalova YA. When Trust Is Not Enough: A Serial Mediation Model Explaining the Effect of Race Identity, eHealth Information Efficacy, and Information Behavior on Intention to Participate in Clinical Research. *Health education & behavior : the official publication of the Society for Public Health Education* 2018; 45: 1036-1042. DOI: doi:<https://dx.doi.org/10.1177/1090198118757822>.

69.	Voytek CD, Jones KT and Metzger DS. Selectively willing and conditionally able: HIV vaccine trial participation among women at "high risk" of HIV infection. *Vaccine* 2011; 29: 6130-6135. DOI: doi:<https://dx.doi.org/10.1016/j.vaccine.2011.06.064>.

70.	White DB, Koehly LM, Omogbehin A, et al. African Americans' responses to genetic explanations of lung cancer disparities and their willingness to participate in clinical genetics research. *Genetics in Medicine* 2010; 12: 496-502. DOI: doi:<https://dx.doi.org/10.1097/GIM.0b013e3181e5e513>.

71.	Williams MM, Scharff DP, Mathews KJ, et al. Barriers and facilitators of African American participation in Alzheimer disease biomarker research. *Alzheimer Disease and Associated Disorders* 2010; 24: S24-S29. DOI: doi:<https://dx.doi.org/10.1097/WAD.0b013e3181f14a14>.

72.	Williams MT, Beckmann-Mendez DA and Turkheimer E. Cultural barriers to African American participation in anxiety disorders research. *Journal of the National Medical Association* 2013; 105: 33-41. DOI: doi:<http://dx.doi.org/10.1016/S0027-9684(15)30083-3>.
