# Supplementary material for: A systematic review and meta synthesis of barriers and facilitators to Black women’s participation in healthcare research
Source: BMC Med Res Methodol. 2026 Jul 21;26:222. doi: 10.1186/s12874-026-02953-7 (PMC13673856; doi:10.1186/s12874-026-02953-7)
Supplement: Supplementary file 5 [file 12874_2026_2953_MOESM5_ESM.docx]

**Additional file 5. Supporting Evidence for key findings.**

Details for each of the 5 key themes and sub-themes are presented in the table below.

## Main Theme 1: Historical and Contemporary Drivers of Mistrust in Research Participation

| **Theme and definition: Historical and Contemporary Drivers of Mistrust in Research Participation** | **References** | **Supporting evidence** |
| --- | --- | --- |
| 1.1 (Mis)Trust in medicine, research(ers) and institutions   *Many studies reported deep-rooted mistrust of healthcare systems, healthcare professionals, research(ers) and public institutions more broadly among Black communities. This was often shaped by both personal experiences and collective historical trauma.* | n=45 ^1-45^ | - “You know, just the history with the Tuskegee experiment. The history with slavery. How people used to practice maternity procedures on Black women without any type of anaesthesia. I’m not 100% confident that that mentality is extinct in this country. So, I’m very hesitant to contribute and volunteer my body.”^2^ - “So, now it comes down to the health professionals that are conducting these studies. Have they been manipulated to not lure but encourage these patients to participate to measure the effectiveness of the drug? Or are they being manipulated because there’ll be some kind of financial gain in there for them? Now, we have to put our trust in the health professionals that are conducting this, and I would like to believe that most of them would do this for the good of the patient or for medicine, not just for financial gains because that would be awful.”^17^ - “If it is supposed to be confidential…how can they be sure?… They are probably afraid that it is something that might entrap them, or be used against them, or they are suspicious; no trust… That's because they don't know who is running it. Suspicious like wondering why they are calling them or why they are here and what is it going to be about.”^40^ |
| 1.2 Building Trust through cultural sensitivity and engagement   *Providing professional, accessible, transparent and detailed information about the research with culturally competent researchers who undertook meaningful community engagement could build trust in research(ers).* | n=36^1-7, 9-11, 13-15, 18-20, 23, 24, 27, 28, 33, 36, 39, 40, 42, 44, 46-55^ | - “If you know what your rights are- part of the reason we were taken advantage of before is that we didn’t know what our rights were, but we have to teach ourselves and know what those things are.”^1^ - “I think they should start off by saying, ‘This is a safe space. I’ve done my research; I know what Black women go through and I’m doing this study, or I want to hear from you because I genuinely care.’ I feel like just saying that alone makes us feel much more comfortable to talk and not just, ‘Hi, I’m here to interview you about Black women’s sexual health.’”^14^ - “A lot of times we’re not at the table. . . But if there is an understanding from the beginning all the way through, then I think you have more buy-in, you have people that are more committed to it. And then they can talk from a place of how important and valuable it is for a person to be involved in that research.”^10^ |

## Main Theme 2: Social and Relational Determinants of Engagement

| **Theme and definition: Social and Relational Determinants of Engagement** | **References** | **Supporting evidence** |
| --- | --- | --- |
| 2.1 Quality relationships with professionals  *Positive interactions with research delivery teams and healthcare professionals are key to supporting recruitment and retention of research participants. For some, similarity between researchers and participants can be additionally beneficial for supporting participation.* | n=37^1, 3-12, 14, 15, 17, 20, 22, 24, 26, 27, 29, 32, 34-36, 39, 42, 48, 54-63^ | - “I love the staff. The staff welcomed me. I felt comfortable and safe. I could talk to them about what’s going on with me. They were more like my listeners, not just only an employee, and they allowed me to be myself, and they treated me with respect, regardless of what my past was. I felt comfortable.” Black women, 40-50y/o ^58^ - “If you weren’t Black, I wouldn’t have done this. If you weren’t a woman, I wouldn’t have done this. Representation matters. For me, it’s like a marker of safety. I feel that internalized guilt around what it could mean if I talk about my pain somehow is eased because you’re Black. And it may be fucked up, but I feel like it makes me feel less guilty. Although everybody’s experience is different, I feel like community trauma matters.” Black women^9^ - “There’s no way that I think anyone would have surgery if you just talked to a nurse, to a secretary...and didn’t meet that doctor. I don’t know why in a research situation the research doctor doesn’t think a person wants to talk to him or her. I want to talk to the person that’s doing it on me. . . . I need to eyeball you. I need to see if we can connect. If I can’t connect with you in conversation, I can’t allow you to experiment on my body.”^10^ |
| 2.2 Social, cultural and familial influences  *Hearing about research from a trusted source, whether a known healthcare professional, family/friend, community organisation or prominent community figure can influence research participation positively or negatively, depending upon the viewpoint of that figure.* | n=40^1, 4-7, 9, 12-15, 17, 20, 22-24, 28, 29, 32, 33, 35, 36, 38-41, 44, 45, 49, 50, 53, 54, 56-58, 60, 62, 64-67^ | - “Most of us get info and are motivated to do things from the pulpit.”^1^ - “My mom is very supportive of me. Even though I’m 50, my mom still thinks I’m ﬁve...She didn’t want me to do no kinds of clinical trial at all, because like she said, this is the way she put it...I don’t want...you to be no guinea pig...She say you know you’re too fragile, I don’t want nothing to happen to you...so her answer was no. But like I said, my reason was the timing. But my mom is my support, so...I take to heart what she has to say.”^57^ - “Through a friend. She told me about it. She gave me the phone number to call, and I asked her what it was about. She said it was related to alcohol. She said she knew I drank, so I found out through a friend.” Black women, 50-60 y/o^58^ |

## Main Theme 3: Altruism and Personal Interest as Drivers of Research Participation

| **Theme and definition: Altruism and Personal Interest as Drivers of Research Participation** | **References** | **Supporting evidence** |
| --- | --- | --- |
| 3.1 Research to improve the outcomes of the wider population   *For those motivated to take part in research because they viewed it as an altruistic social good,* *this was often done with the hope that it would make things better for their community or future generations, advance science, improve medical interventions and save lives.* | n=36^1, 2, 5, 6, 8, 10, 12, 15, 16, 18, 21, 22, 24, 25, 28, 30-33, 35, 36, 38, 39, 43-45, 50, 51, 53, 57, 60, 62, 65, 68-70^ | - “I also believe in giving back. I believe whatever you have, you only can keep it [recovery] if you give it away. I don’t want anyone to go through what I did if they can help it. When you have it what do you do when you come home, I’m giving back. You are getting firsthand knowledge from me of my experience in prison, coming home . . . hopefully this information can help someone.”^18^ - “If I can do something to help prolong somebody else's life, even though I might leave mine in the process, I would do it.”^30^ - Participants described both moral duty and pragmatic beliefs about participating in research. One explained, “Nothing will change until at least one person starts.”^39^ |
| 3.2 Research to improve the outcomes of Black women   *Recognising the underrepresentation of Black women in research and the desire to improve this in order to improve the lives of people like them could be a strong motivator for Black women taking part in research.* | n=23^1, 5, 6, 9, 10, 14, 16, 19, 22, 34-37, 39, 42, 44, 46, 50, 51, 62, 68, 70, 71^ | - “Because people of color are consumers of medicine, we’re going to need that [knowledge]. And if you’ve only tested something [on] a White male between the ages of 18 and 25, what does that mean for my mother or my grandmother, who didn’t fit into that category? So, I’ve always felt very strongly about representation. . . that’s the primary reason [for research participation].” Black female participant^10^ - “I was interested in participating because I feel like my voice is never heard. I’ve participated in many women abuse groups and on average typically I may be the only one in the group or maybe there might be someone else of colour but not speciﬁcally Black females and sometimes I felt like, you know, while abuse is abuse, the experiences are different in the way that it’s perceived by others as well.”^9^ - “I see stuff and I just try to give my input, especially if you are talking about Black families…I just want people to realize that there is a disparity in the services you receive based upon where you live and your race.”^35^ |
| 3.3 The value of renumeration, incentives and benefits   *A range of benefits could motivate participants to take part in research including financial incentives, access to treatment, learning opportunities, socialising and therapeutic experiences.* | n=34^2, 6-8, 10, 13, 15, 16, 18, 21-23, 25, 29, 30, 32, 33, 35, 38-41, 43-45, 50-52, 54, 55, 58, 59, 70, 71^ | - “Well, I had issues at the moment, I needed a couple of dollars too. So it was – they helped me and I helped them. I gave them what they needed and they gave me some money.” (Black woman, 30-40 years old)^58^ - “With these studies that are prevalent now and targeted for minority environments, the way I see it is that [it] affords them an opportunity to get medical attention that perhaps they couldn’t have gotten earlier because they did not have proper medical attention.”^33^ - “Sometimes you ask the questions, and maybe its triggering something, but you might need that trigger so it can pull something out of you and release something so you are not walking around holding onto that, angry but you don’t know why you are angry . . . You really need to get it out, speak it to someone who will not judge you, a listening ear. You never know what kinds of questions you need asked to just trigger something to get it out”^18^ |

## Main theme 4: Weighing the Burdens and Risks of Research Participation

| **Theme and definition: Weighing the Burdens and Risks of Research Participation** | **References** | **Supporting evidence** |
| --- | --- | --- |
| 4.1 Safety concerns with medical research  *Safety concerns, including potential side effects or unexpected consequences of taking part in medical research were the main burden of participation highlighted.* | n=32^1-3, 10-13, 15-17, 21, 24, 29, 30, 32, 33, 35, 36, 38, 39, 41-45, 49, 52, 54, 57, 62, 70, 72^ | - “[...] my first concern would be how is that going to interact with what I’m already taking? How is that going to interact with the current medication that I’m on that’s keeping me alive?”^2^ - [M]essages related to the theme “Everything has been a trial” provides a perhaps unconsidered perspective of clinical trials. One participant noted, “This is an idea of what the process looks like. It lets me know that I’m not the first person, or before it even gets to me, before it even gets to humans, it’s been tested for a minimum of 6 years, which is important. That makes me feel not like a guinea pig.”^12^ - “I would be leery of that just because now there’s an unborn child, and you don’t know all the risks. Depending on the research, and if it’s something where you’re just asking questions or doing things like that - but if we’re testing drugs or procedures - things like that - that puts in jeopardy two people instead of one, so…”^49^ |
| 4.2 Practical/logistical considerations  *The inconvenience of taking part in research often involved practical consideration for the participant such as the time and location of the study, disruption to daily life and taking time off work.* | n=29^4, 7, 12, 13, 15, 17, 19, 22, 23, 25, 28-30, 33, 35, 38, 39, 42-44, 46, 50-53, 55, 56, 58, 72^ | - “Actually, it was a very convenient study so it wasn’t taking me out of nothing else I was really doing. I’d just go to the study, and then I’d go do whatever I was doing. It was very convenient. The time, I got to meet new people – you know, a couple people in there. It’s not far from my house, so it’s convenient.” (female, 30–40 years old, Black woman) ^58^ - “It’s expensive so financial, and your time that you have to take off. We were out, and then she had complications, so she was out of school for a year. We were at home for a year.”^29^ - “…how far I may have to go, what days would I be home, what time you want me there…Convenience, that’s all that matters.” 28 y.o. woman^38^ |
| 4.3 Languages/accessibility barriers  *A lack of clear and accessible materials in different languages and formats (e.g. physical copies, online resources, videos) is key to reducing the burden of participation on those taking part and attracting a diverse sample.* | n=10^15, 23, 28, 34-36, 39, 42, 50, 53^ | - “…it's a huge one because…I think we tend to think that interpreter services do a great job, even me, myself, has been in a place where a family member had an interpreter and understanding my own language, native language, and English, there were times where I saw that there was discrepancy, you know, that the person wasn't necessarily interpreting things correctly or in away that the person could understand it.” (Eritrean community leader)^28^ - “One of the moms of [my child]’s classmates was talking about [the research]…She’s like, ‘They sent home a thing and I didn’t know what [study flyer] meant’…If [researchers] do send home something about, ‘There’s a study you can participate in’ or ‘There’s a resource’ or ‘There’s a group,’ the language has to be basic, and there can’t be too many words because we’re dealing with people who are not necessarily literate…”^35^ |

## Main theme 5: Structural and Informational Foundations of Research Participation

| **Theme and definition: Structural and Informational Foundations of Research Participation** | **References** | **Supporting evidence** |
| --- | --- | --- |
| 5.1 Reaching Black communities  *Contributing to the widespread lack of awareness about research opportunities was, in part at least, due to how researchers try to engage Black communities.* | n=11^2, 4, 14, 15, 20, 33, 56, 60, 62-64^ | - “I’m not aware of opportunities. I would feel more comfortable if I could go on Meharry’s website or you know Vanderbilt website or even if it was a hospital website, you know, St. Thomas Midtown or whatever and see what’s going on. And if there was something I wanted to sign up for I would feel comfortable doing that as long as the information was, you know, laid out”^64^ - [A]ll of the focus group participants agreed that posting information at churches would be a good avenue for informing people about participating in clinical studies, other than the doctor’s ofﬁce.^20^ - “I do a lot of social media stuff. I would stick with Instagram specifically, because I think if you’re looking for a specific age range, the college age range is mostly on Instagram, but as far as finding people and reaching out to people, it would be more through connections” ^14^ |
| 5.2 Limited health and research literacy  *It was recognised that there needed to be efforts to increase health literacy and educate populations on research, health conditions and treatments to increase accessibility.* | n=27^1-3, 5, 6, 8, 10-12, 15, 17, 19, 21, 25, 26, 28, 29, 32, 36, 37, 41, 42, 44, 46, 54, 55, 57^ | - “There are certain areas, certain pockets throughout the country that have poor health literacy rates. If my health literacy is piss poor, do you really think I know something about research? And do you really think I’m gone trust it.”^2^ - “Clinical trial we know is a trial and some people get the A, B, and C drug and some people get the A, B, and sugar drug. So I think that’s our biggest fear with doing all of this and then I’m not getting the real deal (...). Getting the placebo or whatever it is (...). I mean, the only thing is, and I understand how Black people don’t want to do trials, is because I know that to prepare for a trial, it’s a lot of work and it’s very regimented that you have to do all these things. And then just to find out at the end that you didn’t get the actual drug. You know, that’s pretty discouraging.”^12^ - “You know there is a lack of awareness or lack of information or lack of education, so they don’t know the questions to ask because the only thing that they know is what they’ve heard. And I think there’s this….lack of understanding about what’s going on and a lot of people have preconceived notions about what clinical trials are about or what happens as a result of clinical trials….and I think we have to go back and do a lot of education”^21^ |

## References

1.	Bardach SH, Yarbrough M, Walker C, et al. Insights From African American Older Adults on Brain Health Research Engagement: "Need to See the Need". *Journal of Applied Gerontology* 2021; 40: 201-208. DOI: doi:<https://dx.doi.org/10.1177/0733464820902002>.

2.	Barre I, Cunningham-Erves J, Moss J, et al. Motivators and Barriers to COVID-19 Research Participation at the Onset of the COVID-19 Pandemic in Black Communities in the USA: an Exploratory Study. *Journal of Racial & Ethnic Health Disparities* 2023; 10: 2890-2899. DOI: doi:10.1007/s40615-022-01466-5.

3.	Bass SB, Wolak C, Greener J, et al. Using perceptual mapping methods to understand gender differences in perceived barriers and benefits of clinical research participation in urban minority HIV+ patients. *AIDS Care* 2016; 28: 528-536. DOI: doi:10.1080/09540121.2015.1112352.

4.	Blanchet R, Sanou D, Nana CP, et al. Strategies and Challenges in Recruiting Black Immigrant Mothers for a Community-Based Study on Child Nutritional Health in Ottawa, Canada. *Journal of Immigrant & Minority Health* 2017; 19: 367-372. DOI: doi:<https://dx.doi.org/10.1007/s10903-016-0536-6>.

5.	Brewer LPC, Pasha M, Seele P, et al. Overcoming Historical Barriers: Enhancing Positive Perceptions of Medical Research Among African Americans Through a Conference-Based Workshop. *Journal of General Internal Medicine* 2021; 36: 2547-2554. DOI: doi:<https://dx.doi.org/10.1007/s11606-021-06736-2>.

6.	Buseh AG, Stevens PE, Millon-Underwood S, et al. Community leaders' perspectives on engaging African Americans in biobanks and other human genetics initiatives. *Journal of Community Genetics* 2013; 4: 483-494. DOI: doi:<https://dx.doi.org/10.1007/s12687-013-0155-z>.

7.	Cudjoe J, Turkson-Ocran R-A, Ezeigwe AK, et al. Recruiting African Immigrant Women for Community-Based Cancer Prevention Studies: Lessons Learned from the AfroPap Study. *Journal of Community Health* 2019; 44: 1019-1026. DOI: doi:10.1007/s10900-019-00677-y.

8.	Disbrow EA, Arnold CL, Glassy N, et al. Alzheimer Disease and Related Dementia Resources: Perspectives of African American and Caucasian Family Caregivers in Northwest Louisiana. *Journal of Applied Gerontology* 2021; 40: 209-219. DOI: doi:10.1177/0733464820904568.

9.	Duhaney P. Fostering Change: Black Women's Motivations for Participating in Intimate Partner Violence Research. *Affilia: Feminist Inquiry in Social Work* 2024; 39: 265-284. DOI: doi:10.1177/08861099231187861.

10.	Eliacin J, Hathaway E, Wang S, et al. Factors influencing the participation of Black and White Americans in Alzheimer's disease biomarker research. *Alzheimer's and Dementia: Diagnosis, Assessment and Disease Monitoring* 2022; 14. DOI: doi:<https://dx.doi.org/10.1002/dad2.12384>.

11.	Evans K, Lewis M, Hudson S, et al. The role of health literacy on African American and Hispanic/Latino perspectives on cancer clinical trials. *Journal of Cancer Education* 2012; 27: 299-305. DOI: doi:10.1007/s13187-011-0300-5.

12.	Fairley R, Lillard JW, Jr., Berk A, et al. Increasing Clinical Trial Participation of Black Women Diagnosed with Breast Cancer. *Journal of Racial & Ethnic Health Disparities* 2024; 11: 1701-1717. DOI: doi:<https://dx.doi.org/10.1007/s40615-023-01644-z>.

13.	Fincham D, Kagee A and Swartz L. Inhibitors and facilitators of willingness to participate (WTP) in an HIV vaccine trial: Construction and initial validation of the Inhibitors and Facilitators of Willingness to Participate Scale (WPS) among women at risk for HIV infection. *AIDS Care - Psychological and Socio-Medical Aspects of AIDS/HIV* 2010; 22: 452-461. DOI: doi:<https://dx.doi.org/10.1080/09540120903202939>.

14.	Frederick H, Wade J, Parker S, et al. Understanding Openness to Involvement in Sexual Health Care Research: Narratives from a Sample of Black College Women in the United States. *Journal of Sex Research* 2022; 59: 1122-1132. DOI: doi:<https://dx.doi.org/10.1080/00224499.2021.1994515>.

15.	Frierson GM, Pinto BM, Denman DC, et al. Bridging the Gap: Racial concordance as a strategy to increase African American participation in breast cancer research. *Journal of Health Psychology* 2019; 24: 1548-1561. DOI: doi:<https://dx.doi.org/10.1177/1359105317740736>.

16.	Gatny HH and Axinn WG. Willingness to participate in research during pregnancy: Race, experience, and motivation. *Field Methods* 2012; 24: 135-154. DOI: doi:<https://dx.doi.org/10.1177/1525822X11419819>.

17.	Granda-Cameron C, McLean Florence Y, Whitfield-Harris L, et al. Perceptions of Clinical Trial Participation in African American Cancer Survivors and Caregivers. *Oncology Nursing Forum* 2022; 49: 113-124. DOI: doi:10.1188/22.ONF.113-124.

18.	Gunn AJ, Hardesty M, Overstreet N, et al. "Every time I tell my story I learn something new": Voice and inclusion in research with Black women with histories of substance use and incarceration. *Criminology & Criminal Justice: An International Journal* 2022; 22: 505-524. DOI: doi:<https://dx.doi.org/10.1177/17488958211005820>.

19.	Gwizdala KL, Pugh EA, Carter L, et al. Impact of COVID-19 Pandemic on Research Participation among Older African Americans. *Alzheimer Disease and Associated Disorders* 2022; 36: 350-353. DOI: doi:<https://dx.doi.org/10.1097/WAD.0000000000000512>.

20.	Haynes-Maslow L, Godley P, Dimartino L, et al. African American women's perceptions of cancer clinical trials. *Cancer Medicine* 2014; 3: 1430-1439. DOI: doi:<https://dx.doi.org/10.1002/cam4.284>.

21.	Hughes TB, Varma VR, Pettigrew C, et al. African Americans and Clinical Research: Evidence Concerning Barriers and Facilitators to Participation and Recruitment Recommendations. *The Gerontologist* 2017; 57: 348-358. DOI: doi:<https://dx.doi.org/10.1093/geront/gnv118>.

22.	James DC, Harville C, 2nd, Whitehead N, et al. Willingness of African American Women to Participate in e-Health/m-Health Research. *Telemedicine Journal & E-Health* 2016; 22: 191-197. DOI: doi:<https://dx.doi.org/10.1089/tmj.2015.0071>.

23.	Johnson CK, Leung MM, Ma GX, et al. Effective Recruitment Strategies Utilized to Examine Dietary Practices of Blacks in New York City in the Midst of the COVID-19 Pandemic. *Journal of Racial & Ethnic Health Disparities* 2024; 11: 764-772. DOI: doi:10.1007/s40615-023-01559-9.

24.	Kapiriri L, Tharao W, Muchenje M, et al. How acceptable is it for HIV positive African, Caribbean and Black women to provide breast milk/fluid samples for research purposes? *BMC Research Notes* 2017; 10: 7. DOI: doi:<https://dx.doi.org/10.1186/s13104-016-2326-6>.

25.	Lang R, Kelkar VA, Byrd JR, et al. African American participation in health-related research studies: indicators for effective recruitment. *Journal of public health management and practice : JPHMP* 2013; 19: 110-118. DOI: doi:.

26.	Lincoln KD, Chow T, Gaines BF, et al. Fundamental causes of barriers to participation in Alzheimer's clinical research among African Americans. *Ethnicity & health* 2021; 26: 585-599. DOI: doi:<https://dx.doi.org/10.1080/13557858.2018.1539222>.

27.	Morgan SE, Harrison TR, Wright KO, et al. The role of perceived expertise and trustworthiness in research study and clinical trial recruitment: Perspectives of clinical research coordinators and African American and Black Caribbean patients. *PLOS One* 2023; 18. DOI: doi:<https://dx.doi.org/10.1371/journal.pone.0275770>.

28.	O'Connor MR, Adem A and Starks H. East African Perceptions of Barriers/Facilitators for Pediatric Clinical Research Participation and Development of the Inclusive Research Model. *Journal of Pediatric Nursing* 2018; 42: 104-110. DOI: doi:10.1016/j.pedn.2018.05.005.

29.	Omondi NA, Ferguson SES, Majhail NS, et al. Barriers to hematopoietic cell transplantation clinical trial participation of African American and black youth with sickle cell disease and their parents. *Journal of Pediatric Hematology/Oncology* 2013; 35: 289-298. DOI: doi:<https://dx.doi.org/10.1097/MPH.0b013e31828d5e6a>.

30.	Poteat T, Aqil A, Corbett D, et al. "I would really want to know that they had my back": Transgender women's perceptions of HIV cure-related research in the United States. *PLoS ONE [Electronic Resource]* 2020; 15: e0244490. DOI: doi:<https://dx.doi.org/10.1371/journal.pone.0244490>.

31.	Reddy A, Amarnani A, Chen M, et al. Privacy Concerns About Personal Health Information and Fear of Unintended Use of Biospecimens Impact Donations by African American Patients. *Journal of Cancer Education* 2020; 35: 522-529. DOI: doi:10.1007/s13187-019-01491-9.

32.	Rivers DA, Pal T, Vadaparampil ST, et al. A community-academic partnership to explore informational needs of African American women as a primer for cancer clinical trial recruitment. *Ethnicity & Health* 2019; 24: 679-693. DOI: doi:<https://dx.doi.org/10.1080/13557858.2017.1367762>.

33.	Rollins L, Sy A, Crowell N, et al. Learning and action in community health: Using the health belief model to assess and educate African American community residents about participation in clinical research. *International Journal of Environmental Research and Public Health* 2018; 15. DOI: doi:<https://dx.doi.org/10.3390/ijerph15091862>.

34.	Scharf DP, Mathews KJ, Jackson P, et al. More than Tuskegee: Understanding mistrust about research participation. *Journal of Health Care for the Poor and Underserved* 2010; 21: 879-897. DOI: doi:<https://dx.doi.org/10.1353/hpu.0.0323>.

35.	Shaia WE, Nichols HM, Dababnah S, et al. Brief Report: Participation of Black and African-American Families in Autism Research. *Journal of Autism and Developmental Disorders* 2020; 50: 1841-1846. DOI: doi:<https://dx.doi.org/10.1007/s10803-019-03926-0>.

36.	Sneed RS, Mason M, Williams JN, et al. Using Critical Race Theory to Understand Trial Participation Among Black Individuals With Systemic Lupus Erythematosus: A Qualitative Study of Patients and Caregivers. *Arthritis Care and Research* 2021; 73: 1387-1395. DOI: doi:<https://dx.doi.org/10.1002/acr.24635>.

37.	Somayaji D and Cloyes KG. Cancer fear and fatalism: How African American participants construct the role of research subject in relation to clinical cancer research. *Cancer Nursing* 2015; 38: 133-144. DOI: doi:<https://dx.doi.org/10.1097/NCC.0000000000000144>.

38.	Voytek CD, Jones KT and Metzger DS. Selectively willing and conditionally able: HIV vaccine trial participation among women at "high risk" of HIV infection. *Vaccine* 2011; 29: 6130-6135. DOI: doi:<https://dx.doi.org/10.1016/j.vaccine.2011.06.064>.

39.	Williams MM, Scharff DP, Mathews KJ, et al. Barriers and facilitators of African American participation in Alzheimer disease biomarker research. *Alzheimer Disease and Associated Disorders* 2010; 24: S24-S29. DOI: doi:<https://dx.doi.org/10.1097/WAD.0b013e3181f14a14>.

40.	Williams MT, Beckmann-Mendez DA and Turkheimer E. Cultural barriers to African American participation in anxiety disorders research. *Journal of the National Medical Association* 2013; 105: 33-41. DOI: doi:<http://dx.doi.org/10.1016/S0027-9684(15)30083-3>.

41.	Berg KA, Marbury M, Whaley MA, et al. Perspectives of African American couples regarding participation in trials of treatments for obstructive sleep apnea. *Journal of the National Medical Association* 2025; 117: 449-457. DOI: <https://doi.org/10.1016/j.jnma.2025.08.106>.

42.	Bontemps-Jones JE, McCullough LE, Kirkland EG, et al. Beyond Tuskegee: A contemporary qualitative assessment of barriers to research participation among Black women. *Cancer* 2025; 131: e35648. DOI: <https://doi.org/10.1002/cncr.35648>.

43.	Dobreva I, Kalam S, Roche M, et al. Understanding barriers and facilitators to participation in Parkinson's research in Black communities in the UK. *Journal of Parkinson’s Disease* 2025; 15: 1035-1039. DOI: 10.1177/1877718x251351990.

44.	Gebremedhen T, Campbell AR, Magagula P, et al. Understanding Research Participation Experiences Among Persons Identifying as African, Caribbean, and Black in British Columbia. *Prog Community Health Partnersh* 2025; 19: 169-178. DOI: 10.1353/cpr.2025.a965354.

45.	Malova E, Jia X, Harrison TR, et al. Tensions Within Incentives, Altruism, and Trust in Clinical Trial Participation: Perspectives From African American and Black Caribbean Patients and Clinical Research Coordinators Who Recruit Them. *Journal of Racial and Ethnic Health Disparities* 2025. DOI: 10.1007/s40615-025-02701-5.

46.	Colon-Otero G, Albertie M, Lesperance M, et al. A pilot program in collaboration with African American churches successfully increases awareness of the importance of cancer research and participation in cancer translational research studies among African Americans. *Journal of Cancer Education* 2012; 27: 294-298. DOI: doi:10.1007/s13187-011-0288-x.

47.	Cox LS, Faseru B, Mayo MS, et al. Design, baseline characteristics, and retention of African American light smokers into a randomized trial involving biological data. *Trials* 2011; 12: 22-22. DOI: doi:10.1186/1745-6215-12-22.

48.	Morgan SE, Harrison TR, Wright KO, et al. Reducing Health Disparities Among African American and Black Caribbean Patients by Improving the Communication Practices of Clinical Research Coordinators. *Health communication* 2024; 39: 1298-1309. DOI: doi:<https://dx.doi.org/10.1080/10410236.2023.2211740>.

49.	Ngui EM, Warner TD and Roberts LW. Perceptions of African-American health professionals and community members on the participation of children and pregnant women in genetic research. *Public Health Genomics* 2014; 17: 23-32. DOI: doi:<https://dx.doi.org/10.1159/000355359>.

50.	Occa A, Francis DB, Grumbein A, et al. Improving African Americans' Intentions to Enroll in a Health Registry: Message Development and Evaluation. *Health Communication* 2023; 38: 2884-2893. DOI: doi:10.1080/10410236.2022.2124061.

51.	Overstreet NM, Okuyan M and Fisher CB. Perceived Risks and Benefits in IPV and HIV Research: Listening to the Voices of HIV-Positive African American Women. *Journal of Empirical Research on Human Research Ethics* 2018; 13: 511-524. DOI: doi:<https://dx.doi.org/10.1177/1556264618797557>.

52.	Pugh E, Stewart J, Carter L, et al. Beliefs, Understanding, and Barriers Related to Dementia Research Participation Among Older African Americans. *Alzheimer Disease & Associated Disorders* 2022; 36: 52-57. DOI: doi:10.1097/WAD.0000000000000476.

53.	Chung JE, Cramer EM, Uche S, et al. Engaging Minoritized Communities in Clinical Trials Through Social Media: Recommendations from Community-Based Participatory Research. *Health Communication* 2025; 40: 2696-2704. DOI: 10.1080/10410236.2025.2476684.

54.	Olakunde BO, Idemili-Aronu N, Friebel-Klingner TM, et al. Optimizing Recruitment and Retention in Cancer Clinical Trials in Low-Resource Settings: Barriers and Facilitators From Nigerian Provider's Perspectives. *JCO Glob Oncol* 2025; 11: e2400308. 20250213. DOI: 10.1200/go-24-00308.

55.	Shiyanbola OO, Wen M-J and Maurer MA. Incorporating health literacy principles into the adaptation of a methods motivational interviewing approach for enrolling black adults in a pilot randomized trial. *Scientific Reports* 2025; 15: 4833. DOI: 10.1038/s41598-025-89413-2.

56.	Adams SA, Heiney SP, Brandt HM, et al. A comparison of a centralized versus de-centralized recruitment schema in two community-based participatory research studies for cancer prevention. *Journal of community health* 2015; 40: 251-259. DOI: doi:<https://dx.doi.org/10.1007/s10900-014-9924-9>.

57.	Brown RF, Cadet DL, Houlihan RH, et al. Perceptions of Participation in a Phase I, II, or III Clinical Trial Among African American Patients With Cancer: What Do Refusers Say? *Journal of Oncology Practice* 2013; 9: 287-293. DOI: doi:10.1200/JOP.2013.001039.

58.	Canidate SS, Cook CL, Varma D, et al. Recruitment, experience, and retention among women with HIV and hazardous drinking participating in a clinical trial. *BMC Public Health* 2020; 20: 1169. DOI: doi:<https://dx.doi.org/10.1186/s12889-020-09233-z>.

59.	Fouad MN, Acemgil A, Bae S, et al. Patient Navigation As a Model to Increase Participation of African Americans in Cancer Clinical Trials. *Journal of Oncology Practice* 2016; 12: 554-565. DOI: doi:10.1200/JOP.2015.008946.

60.	Okoruwa P and Ridley-Merriweather KE. Examining recollections of Black women with breast cancer who participated in clinical trials: A grounded practical theory study of patient-provider communication. *Cancer Research Conference* 2022; 83. DOI: doi:<https://dx.doi.org/10.1158/1538-7445.SABCS22-P6-09-03>.

61.	Strekalova YA. When Trust Is Not Enough: A Serial Mediation Model Explaining the Effect of Race Identity, eHealth Information Efficacy, and Information Behavior on Intention to Participate in Clinical Research. *Health education & behavior : the official publication of the Society for Public Health Education* 2018; 45: 1036-1042. DOI: doi:<https://dx.doi.org/10.1177/1090198118757822>.

62.	Ridley-Merriweather KE, Head KJ, Brann M, et al. “We Don’t Get Drugs Targeted for Us:” Applying the Integrated Behavioral Model to Understand Why Black Women Chose to Participate in a Breast Cancer Clinical Trial. *Health Communication* 2025; 40: 2280-2289. DOI: 10.1080/10410236.2024.2448701.

63.	Ridley-Merriweather KE, Okoruwa OP and Vogel K. Examining recollections of Black women with breast cancer who participated in clinical trials: A grounded practical theory study of patient-provider communication. *Contemporary Clinical Trials Communications* 2024; 42: 101403. DOI: <https://doi.org/10.1016/j.conctc.2024.101403>.

64.	Bachour CC, Bachmann GA, Foster DC, et al. Recruitment methods in a clinical trial of provoked vulvodynia: Predictors of enrollment. *Clinical Trials* 2017; 14: 103-108. DOI: doi:<https://dx.doi.org/10.1177/1740774516663461>.

65.	Frew PM, Archibald M, Diallo DD, et al. An extended model of reasoned action to understand the influence of individual- and network-level factors on African Americans' participation in hiv vaccine research. *Prevention Science* 2010; 11: 207-218. DOI: doi:<https://dx.doi.org/10.1007/s11121-009-0162-9>.

66.	Overstreet NM and Cheeseborough T. Examining the Effect of Internalized HIV-Related Stigma on Perceptions of Research Participation among HIV-Positive African American Women. *Translational Issues in Psychological Science* 2020; 6: 223-234. DOI: doi:<https://dx.doi.org/10.1037/tps0000271>.

67.	Princewill CW, Jegede AS, Nordström K, et al. Factors Affecting Women's Autonomous Decision Making In Research Participation Amongst Yoruba Women Of Western Nigeria. *Developing World Bioethics* 2017; 17: 40-49. DOI: doi:10.1111/dewb.12112.

68.	Smith A, Vidal GA, Pritchard E, et al. Sistas Taking a Stand for Breast Cancer Research (STAR) Study: A Community-Based Participatory Genetic Research Study to Enhance Participation and Breast Cancer Equity among African American Women in Memphis, TN. *International Journal of Environmental Research & Public Health [Electronic Resource]* 2018; 15: 18. DOI: doi:<https://dx.doi.org/10.3390/ijerph15122899>.

69.	White DB, Koehly LM, Omogbehin A, et al. African Americans' responses to genetic explanations of lung cancer disparities and their willingness to participate in clinical genetics research. *Genetics in Medicine* 2010; 12: 496-502. DOI: doi:<https://dx.doi.org/10.1097/GIM.0b013e3181e5e513>.

70.	Newton V, Farinu O, Smith H, et al. Speaking Out: Factors Influencing Black Americans’ Engagement in COVID-19 Testing and Research. *Journal of Racial and Ethnic Health Disparities* 2025. DOI: 10.1007/s40615-024-02268-7.

71.	Castillo AG, Jandorf L, Thelemaque LD, et al. Reported benefits of participation in a research study. *Journal of community health* 2012; 37: 59-64. DOI: doi:.

72.	Davis K, Pugh E, Beyl R, et al. Responses to Covid-19 and Research Participation Among Older African Americans. *Journal of Applied Gerontology*; 0: 07334648251369670. DOI: 10.1177/07334648251369670.
