# Supplementary material for: A systematic review and meta synthesis of barriers and facilitators to Black women’s participation in healthcare research
Source: BMC Med Res Methodol. 2026 Jul 21;26:222. doi: 10.1186/s12874-026-02953-7 (PMC13673856; doi:10.1186/s12874-026-02953-7)
Supplement: Supplementary file 6 [file 12874_2026_2953_MOESM6_ESM.docx]

**Additional file 6. Bridges and Barriers Analogy**

Research participation can be depicted as a bridge between Black communities and scientific systems — but one that has been damaged by mistrust, exclusion, and burden, and must be rebuilt with transparency, care, and reciprocity. Each domain either strengthens or weakens the bridge.

| **Main Theme / Domain** | **Illustrative Quote**  (First-order constructs) | **Subtheme**  (Second-order constructs) | **Description / Key Insights** | **Bridge Metaphor Interpretation** | **Practical or Contextualised Examples** |
| --- | --- | --- | --- | --- | --- |
|  |  |  | (Third-order constructs) | |  |
| 1. Historical and Contemporary Drivers of Mistrust in Research Participation | “You know, just the history with the Tuskegee experiment. The history with slavery. How people used to practice maternity procedures on Black women without any type of anesthesia. I’m not 100% confident that that mentality is extinct in this country. So, I’m very hesitant to contribute and volunteer my body.”[1] | 1.1 Mistrust of medicine, research(ers), and institutions | Historical and lived experiences of racism generate deep-rooted mistrust but trust can grow when researchers display competency, integrity and accountability. | Historical trauma weakens the bridge’s foundations, making crossing risky and unappealing but these can be rebuilt through consistent and professional connection. | Hesitation to consent due to historical exploitation; concerns about misuse of personal data or biological samples; reluctance to engage without reassurance about safety and purpose. |
|  | “I think they should start off by saying, ‘This is a safe space. I’ve done my research; I know what Black women go through and I’m doing this study, or I want to hear from you because I genuinely care.’ I feel like just saying that alone makes us feel much more comfortable to talk and not just, ‘Hi, I’m here to interview you about Black women’s sexual health.’”[2] | 1.2 Building trust through cultural sensitivity and engagement | Trust is restored through cultural competency, transparency, consistency, and inclusion. | Anchoring the bridge in community soil can provide a trustworthy foundation, with the bridge further strengthened through collective repair. | Researchers introducing themselves transparently, acknowledging historical harms, using culturally appropriate language, and engaging through trusted community settings (e.g. churches, local groups). |
| 2. Social and Relational Determinants of Engagement | “I love the staff. The staff welcomed me. I felt comfortable and safe. I could talk to them about what’s going on with me. They were more like my listeners, not just only an employee, and they allowed me to be myself, and they treated me with respect, regardless of what my past was. I felt comfortable.” Black women, 40-50y/o [3] | 2.1 Quality relationships with professionals | Empathy and respect from professionals create positive experiences that increase the likelihood of participation. | Relational care can help to pave a smoother pathway for crossing the bridge. | Participants feeling listened to during recruitment conversations; positive interactions with research nurses increasing willingness to enrol and remain in studies. |
|  | “Most of us get info and are motivated to do things from the pulpit.”[4] | 2.2 Social, cultural and familial influences | Family and cultural norms can guide decisions about research participation. | Having companions on the bridge can help to make crossing easier. | Decisions influenced by advice from family members or community leaders; participation encouraged or discouraged through church networks or peer discussions. |
| 3. Altruism and Personal Interest as Drivers of Research Participation | “If I can do something to help prolong somebody else's life, even though I might leave mine in the process, I would do it.”[5] | 3.1 Research to improve the outcomes of the wider population | Altruism motivates people to take part in research for others’ benefit. | The bridge can be seen as a pathway towards collective progress. | Participation motivated by desire to contribute to future treatments or improve outcomes for others with similar conditions. |
|  | “I was interested in participating because I feel like my voice is never heard. I’ve participated in many women abuse groups and on average typically I may be the only one in the group or maybe there might be someone else of colour but not speciﬁcally Black females and sometimes I felt like, you know, while abuse is abuse, the experiences are different in the way that it’s perceived by others as well.”[6] | 3.2 Research to improve the outcomes of Black women | Recognising the lack of diversity and seeking to correct this could be a strong motivator for taking part in research. | It is important to make the bridge an inclusive space for all travelers. | Motivation driven by lack of representation in research and desire to ensure treatments reflect Black women’s needs and experiences. |
|  | “Well, I had issues at the moment, I needed a couple of dollars too. So it was – they helped me and I helped them. I gave them what they needed and they gave me some money.” (Black woman, 30-40 years old)[3] | 3.3 The value of remuneration, incentives, benefits | A range of tangible benefits provide motivation to participate but cannot replace trust. | Incentives can constitute support beams but this is not a substitute for strong foundations. | Reimbursement for travel or childcare enabling participation; financial incentives offsetting lost income from taking time off work. |
| 4. Weighing the Burdens and Risks of Research Participation | “I would be leery of that just because now there’s an unborn child, and you don’t know all the risks. Depending on the research, and if it’s something where you’re just asking questions or doing things like that - but if we’re testing drugs or procedures - things like that - that puts in jeopardy two people instead of one, so…”[7] | 4.1 Safety concerns with medical research | Safety fears act as barriers that deter research participation. | Perceived danger and tolls along the bridge can deter crossing | Concerns about side effects of experimental treatments; reluctance to participate in drug or pregnancy-related studies due to perceived risk. |
|  | “…how far I may have to go, what days would I be home, what time you want me there…Convenience, that’s all that matters.” 28 y.o. woman[8] | 4.2 Practical/ logistical considerations | Time and logistics weigh heavily on decision-making. | If the bridge has restricted access, it can discourage passage. | Difficulty attending study visits due to work schedules, childcare responsibilities, or travel distance; preference for flexible or remote options. |
|  | “I think we tend to think that interpreter services do a great job, even me, myself, has been in a place where a family member had an interpreter and understanding my own language, native language, and English, there were times where I saw that there was discrepancy, you know, that the person wasn't necessarily interpreting things correctly or in away that the person could understand it.” (Eritrean community leader)[9] | 4.3 Language/ accessibility barriers | A lack of clear and accessible services and materials limits access. | A narrow or uneven bridge can exclude some travelers. | Misunderstanding study information due to poor translation; exclusion due to lack of accessible materials (e.g. plain language, different formats). |
| 5. Structural and Informational Foundations of Research Participation | “I’m not aware of opportunities. I would feel more comfortable if I could go on Meharry’s website or you know Vanderbilt website or even if it was a hospital website, you know, St. Thomas Midtown or whatever and see what’s going on. And if there was something I wanted to sign up for I would feel comfortable doing that as long as the information was, you know, laid out”[10] | 5.1 Reaching Black communities | Awareness campaigns must meet people where they are. | Gatekeeping limits access to the bridge. | Limited awareness of opportunities due to reliance on hospital-based recruitment; increased engagement when studies are promoted through community organisations or social media. |
|  | “There are certain areas, certain pockets throughout the country that have poor health literacy rates. If my health literacy is piss poor, do you really think I know something about research? And do you really think I’m gone trust it.”[1] | 5.2 Limited health and research literacy | Education empowers safe, confident decision-making. | Knowledge strengthens bridge stability for future crossings. | Participants declining due to lack of understanding of trials or fear of risks; increased engagement when clear explanations and education are provided. |

**Summary Interpretation (Metaphor Narrative)**

Black women’s participation in research can be understood as a journey across a bridge connecting *communities* and *research systems*.

- **Mistrust, racism, and burden** weaken or narrow the bridge.
- **Transparency, engagement, and literacy** strengthen and widen it.
- **Crossing the bridge safely and willingly** requires co-construction — not just invitation.

1.	Barre, I., et al., *Motivators and Barriers to COVID-19 Research Participation at the Onset of the COVID-19 Pandemic in Black Communities in the USA: an Exploratory Study.* Journal of Racial & Ethnic Health Disparities, 2023. **10**(6): p. 2890-2899.

2.	Frederick, H., et al., *Understanding Openness to Involvement in Sexual Health Care Research: Narratives from a Sample of Black College Women in the United States.* Journal of Sex Research, 2022. **59**(9): p. 1122-1132.

3.	Canidate, S.S., et al., *Recruitment, experience, and retention among women with HIV and hazardous drinking participating in a clinical trial.* BMC Public Health, 2020. **20**(1): p. 1169.

4.	Bardach, S.H., et al., *Insights From African American Older Adults on Brain Health Research Engagement: "Need to See the Need".* Journal of Applied Gerontology, 2021. **40**: p. 201-208.

5.	Poteat, T., et al., *"I would really want to know that they had my back": Transgender women's perceptions of HIV cure-related research in the United States.* PLoS ONE [Electronic Resource], 2020. **15**(12): p. e0244490.

6.	Duhaney, P., *Fostering Change: Black Women's Motivations for Participating in Intimate Partner Violence Research.* Affilia: Feminist Inquiry in Social Work, 2024. **39**(2): p. 265-284.

7.	Ngui, E.M., T.D. Warner, and L.W. Roberts, *Perceptions of African-American health professionals and community members on the participation of children and pregnant women in genetic research.* Public Health Genomics, 2014. **17**: p. 23-32.

8.	Voytek, C.D., K.T. Jones, and D.S. Metzger, *Selectively willing and conditionally able: HIV vaccine trial participation among women at "high risk" of HIV infection.* Vaccine, 2011. **29**(36): p. 6130-5.

9.	O'Connor, M.R., A. Adem, and H. Starks, *East African Perceptions of Barriers/Facilitators for Pediatric Clinical Research Participation and Development of the Inclusive Research Model.* Journal of Pediatric Nursing, 2018. **42**: p. 104-110.

10.	Bachour, C.C., et al., *Recruitment methods in a clinical trial of provoked vulvodynia: Predictors of enrollment.* Clinical Trials, 2017. **14**(1): p. 103-108.
