# Supplementary material for: A systematic review and meta synthesis of barriers and facilitators to Black women’s participation in healthcare research
Source: BMC Med Res Methodol. 2026 Jul 21;26:222. doi: 10.1186/s12874-026-02953-7 (PMC13673856; doi:10.1186/s12874-026-02953-7)
Supplement: Supplementary file 7 [file 12874_2026_2953_MOESM3_ESM.docx]

 **Additional file 3. Data extraction tool.**
